# Pervasive within-host recombination and epistasis as major determinants of the molecular evolution of the foot-and-mouth disease virus capsid

**DOI:** 10.1371/journal.ppat.1008235

**Published:** 2020-01-06

**Authors:** Luca Ferretti, Eva Pérez-Martín, Fuquan Zhang, François Maree, Lin-Mari de Klerk-Lorist, Louis van Schalkwykc, Nicholas D. Juleff, Bryan Charleston, Paolo Ribeca

**Affiliations:** 1 The Pirbright Institute, Woking, Surrey, United Kingdom; 2 Current address: Big Data Institute, Li Ka Shing Centre for Health Information and Discovery, Nuffield Department of Medicine, University of Oxford, Oxford, United Kingdom; 3 South Africa Department of Microbiology and Plant Pathology, University of Pretoria, Pretoria, South Africa; 4 Onderstepoort Veterinary Institute-Transboundary Animal Diseases Programme (OVI-TADP), Onderstepoort, Gauteng, South Africa; University of North Carolina at Chapel Hill, UNITED STATES

## Abstract

Although recombination is known to occur in foot-and-mouth disease virus (FMDV), it is considered only a minor determinant of virus sequence diversity. Analysis at phylogenetic scales shows inter-serotypic recombination events are rare, whereby recombination occurs almost exclusively in non-structural proteins. In this study we have estimated recombination rates within a natural host in an experimental setting. African buffaloes were inoculated with a SAT-1 FMDV strain containing two major viral sub-populations differing in their capsid sequence. This population structure enabled the detection of extensive within-host recombination in the genomic region coding for structural proteins and allowed recombination rates between the two sub-populations to be estimated. Quite surprisingly, the effective recombination rate in VP1 during the acute infection phase turns out to be about 0.1 per base per year, i.e. comparable to the mutation/substitution rate. Using a high-resolution map of effective within-host recombination in the capsid-coding region, we identified a linkage disequilibrium pattern in VP1 that is consistent with a mosaic structure with two main genetic blocks. Positive epistatic interactions between co-evolved variants appear to be present both within and between blocks. These interactions are due to intra-host selection both at the RNA and protein level. Overall our findings show that during FMDV co-infections by closely related strains, capsid-coding genes recombine within the host at a much higher rate than expected, despite the presence of strong constraints dictated by the capsid structure. Although these intra-host results are not immediately translatable to a phylogenetic setting, recombination and epistasis must play a major and so far underappreciated role in the molecular evolution of the virus at all scales.

## Author summary

There are 7 serotypes of Foot-and-Mouth Disease Virus and multiple strains of each serotype. The emergence of new strains can result in widespread outbreaks of disease and requires new vaccines to be developed. The major mechanisms driving variation are thought to be substitutions in the viral genome. Recombination in the capsid-coding region of the virus genome has been described at phylogenetic scales but not thought to play a major role in generating variants. In the current experiment, a co-infection of African buffaloes with closely related sub-populations of viruses allowed us to detect recombination events. For structural protein-coding sequences, the genetic composition of the population is driven by extensive within-host recombination. During the acute infection phase the intra-host recombination rates of 0.1 per base per year are comparable to the typical mutation rates of the virus. The recombination map reveals two strongly linked regions within the VP1 protein-coding sequence. Epistatic interactions between co-evolved mutations in VP1 are caused by intra-host selection at the RNA and protein level and are present both within and between the two regions. Our findings in this experimental setting support a major role for recombination and epistasis in the intra-host evolution of FMDV.

## Introduction

Foot-and-mouth disease virus (FMDV) is a picornavirus of the genus *Aphtovirus* that causes foot-and-mouth disease (FMD), a highly contagious vesicular disease. FMD is one of the most economically important diseases of cloven-hoofed animals [1]. Domestic and wild artiodactyls usually develop viraemia a few days after exposure to FMDV, followed by the appearance of clinical signs of acute infection characterized by vesicles in mouth and feet, which last about a week. In some cases such as African buffaloes, the infection progresses in a subclinical form and the virus can persist for years in carrier animals [2].

The FMDV genome is approximately 8000 nucleotides long and encodes a single open reading frame coding for a leader polypeptide (Lpro) that cleaves itself from the polyprotein, four structural proteins (1A–1D or VP4, VP2, VP3, VP1) and nine non-structural proteins (2A–2C, 3A, 3B1–3B3, 3C, 3D) [3, 4]. The determinants for immunity are mostly found in VP1–VP4, which form the viral capsid.

Mutation rates in the FMDV genome are high, especially in the capsid-coding region. As it is often the case in RNA viruses [5], this is partly due to the lack of proof-reading capabilities of the polymerase. The high substitution rates contribute to the substantial genetic and antigenic variability of the virus. Seven different serotypes—A, O, C, Asia1 and Southern African Territories (SAT) 1/2/3—are known, with a distribution spanning from south-eastern Asia to Africa and South America [6, 7]. SAT serotypes are endemic to Africa, where they circulate mostly among African buffaloes (*Syncerus caffer*).

Inside its animal hosts, the high mutation rates of FMDV may lead to the formation of a viral swarm, i.e. a cloud of similar genotypes differing only by a handful of mutations [8]. This is a typical pattern of intra-host genetic variability in RNA viruses with high mutation rates [5, 9] and is often correlated with a rich quasi-species dynamics [10].

Another important mechanism in the evolution of FMDV genomes is recombination [11–13]. Direct evidences of FMDV recombination date back 40 years ago [14, 15]. Most recombination breakpoints are observed in non-structural proteins. Recombinant capsid-coding sequences have been described [16, 17], but they appear to be much rarer than recombination events in non-structural proteins. Systematic studies [18, 19] have found phylogenetic evidences of extensive recombination among non-structural proteins and only a small number of recombination events within capsid-coding sequences.

Recombination inferred from phylogenetic studies suffers from a strong detection bias. In fact, only events that occur between sufficiently divergent lineages can be detected, and only events that do not disrupt positive epistatic interactions among variants (i.e. events preserving correlated sets of genomic variants that taken together confer an evolutionary advantage to the virus) can generate viral sequences that are fit enough to be observed in samples [19, 20]. In addition, since the capsid proteins are the primary target of the immune response, cross-immunity of viruses with similar capsid-coding sequences could reduce co-infections and therefore recombination.

Within-host studies offer the opportunity to observe recombination in action without any of these biases [21, 22]. Furthermore, intra-host recombination is an interesting subject in itself due to its role in the generation of genetic diversity within hosts [10, 23, 24]. In this respect, one of the best experimental systems for FMDV is arguably represented by infections in African buffalo, since animals of this species are FMDV carriers: after an initial acute phase of the infection, the virus can persist for years in some tissues, albeit at lower levels of replication [2]. In principle, this increases the chances to observe recombination events. The SAT serotypes of the virus are well-adapted to this host and there is evidence that buffaloes contribute to their dissemination [25].

In a recent experiment on African buffaloes infected by FMDV [2], viral sequences from different animals and tissues were generated with a mixture of Sanger and high-throughput sequencing technologies. An interesting feature of this experiment is the subsequent discovery of a strong genetic structure among the viral sequences. It turns out that both the inoculum and the animal samples contain two major viral sub-populations with moderate sequence divergence between them. Our results show that recombinants of these swarms were already present in the inoculum—probably due to previous recombination in buffalo or in culture—and the amount of recombination increased both after the acute phase of the infection and during the persistent phase. Thus this experimental system provides an excellent setup to infer the relative and absolute rates of within-host recombination.

In this paper, we present a detailed analysis of the genomic patterns of recombination in the capsid-coding region. First, we provide a brief explanation of the experimental setup and how it enables us to detect within-host recombination accurately. Then we present some estimates of the absolute recombination rates during the infection process—which turn out to be comparable to the substitution rates—and infer the recombination profile inside the capsid-coding region. Focusing on the VP1 coding region, we show how the linkage disequilibrium (LD) patterns among variants suggest a mosaic structure inside VP1, with two main genetic blocks showing reduced recombination within each block. In addition, these patterns indicate the existence of intra-host epistasis between variants from different sub-populations, with epistatic interactions acting both within and between blocks. Finally, we discuss the evolutionary consequences of our findings both for the intra-host quasi-species dynamics and for the long-term evolution of FMDV.

## Results

### Experimental setup and viral population structure

**Fig 1:**
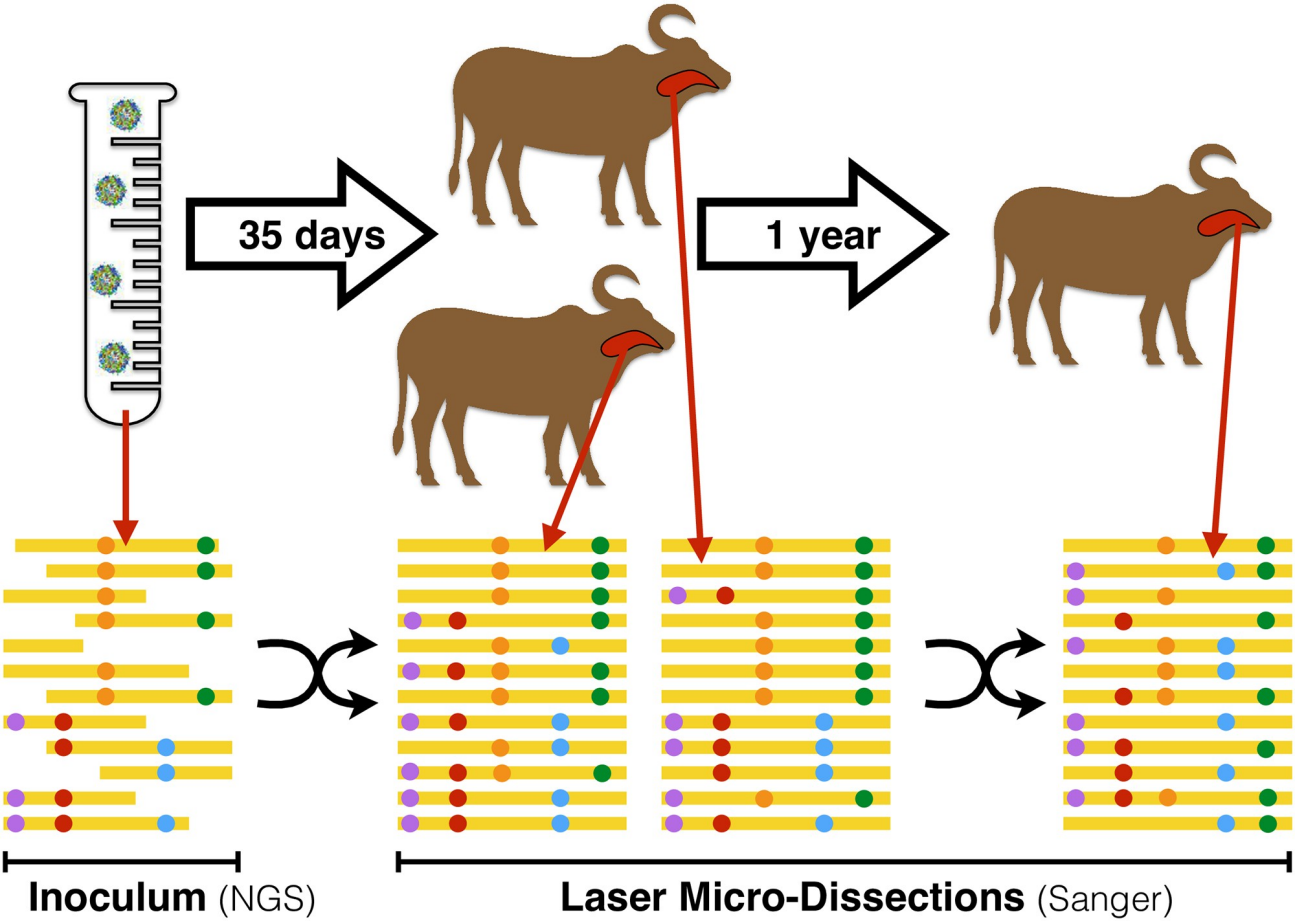
Illustration of the setup for the artificial inoculation and sampling (above) and the sequencing data (below) for the experiment.

African buffaloes (*Syncerus caffer*) were co-infected with the three FMDV Southern African Territories serotypes (SAT1, SAT2, SAT3). The inoculum contained equal titres of SAT1, SAT2, and SAT3 virus, but only SAT1 was found in infected buffaloes one year after infection. The other serotypes play no known role in the dynamics of SAT1 diversity, hence we focus only on this serotype. Deep short-read sequencing data were obtained from the SAT1 inoculum, while Sanger sequences were obtained from the oro-pharingeal tract of three infected buffaloes, as illustrated in Fig 1.

**Fig 2:**
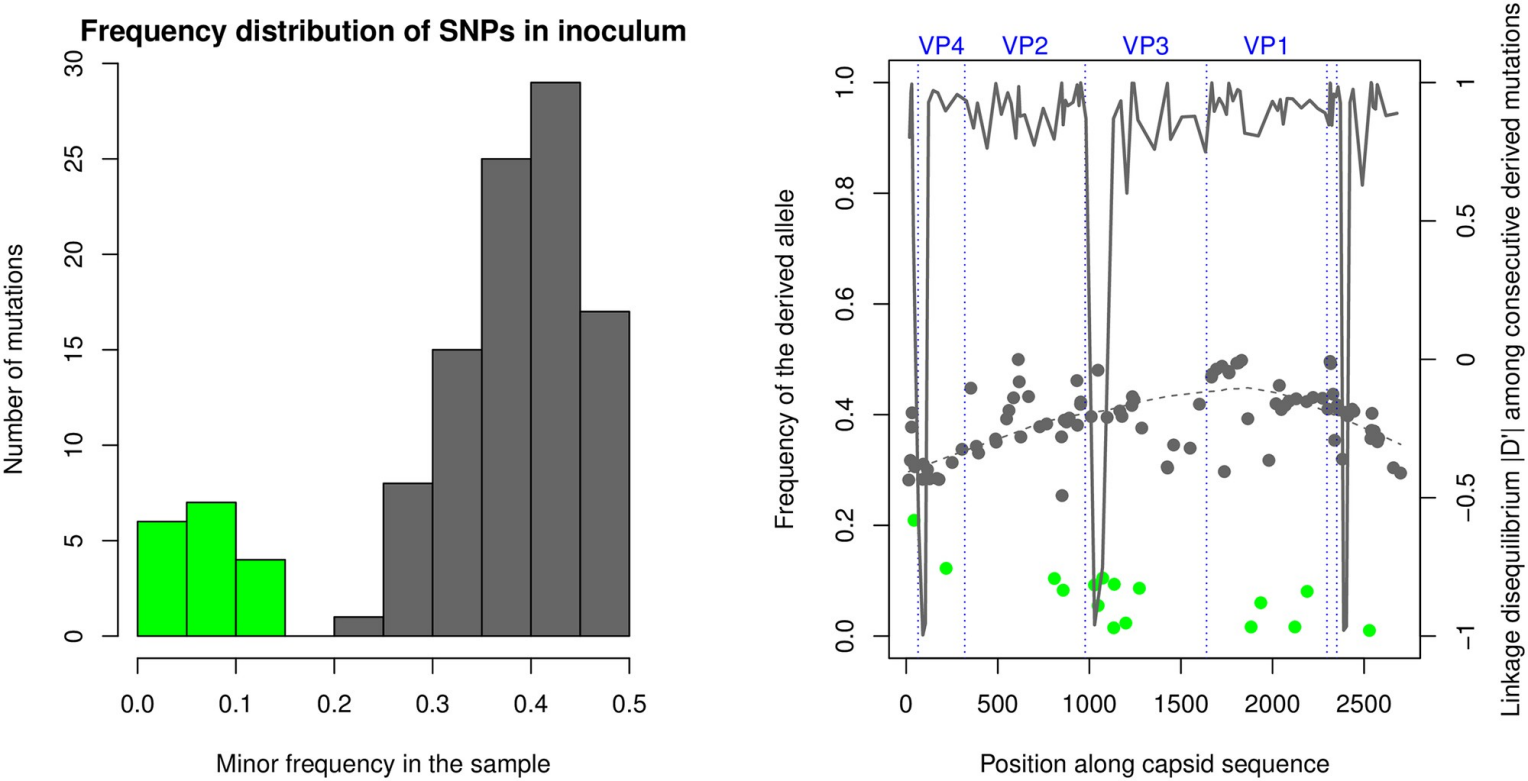
A: Distribution of minor SNV frequencies in the reads from the inoculum. The distribution is clearly bimodal, separating subpopulation-specific SNVs with minor frequency > 0.2 (grey) from intra-swarm ones (green). B: Location and frequency of the derived allele of SNVs, both subpopulation-specific (grey points) and intra-swarm (green points). The dashed line is the LOWESS of subpopulation-specific SNVs. The continuous line shows the linkage disequilibrium *D*′ between pairs of consecutive derived variants.

For the inoculum, a region of about 3 kb containing the capsid-coding region of the SAT1 component was sequenced at high read depth (about 30000 reads per base). Interestingly, an analysis of the nucleotide polymorphisms among the short reads revealed a peculiar distribution of variant frequencies with a strong peak around a frequency of 44%, as illustrated in Fig 2. All the variants around this frequency are in strong linkage disequilibrium, i.e. reads covering two of these polymorphic sites tend to contain either the minor allele or the major allele at both sites.

These results imply that the capsid-coding sequences of SAT1 viruses in the inoculum exhibit a strong sub-population structure, with most nucleotide sequences belonging to one of two major viral swarms (according to the literature in the field previously mentioned, we use the term “viral swarm” to indicate a cloud of similar genotypes differing only by a few mutations). The VP1-coding sequences of the two sub-populations differ by about 3%, much larger than the genetic diversity within each swarm. Hence, the two sub-populations are clearly genetically distinct and separable.

**Fig 3:**
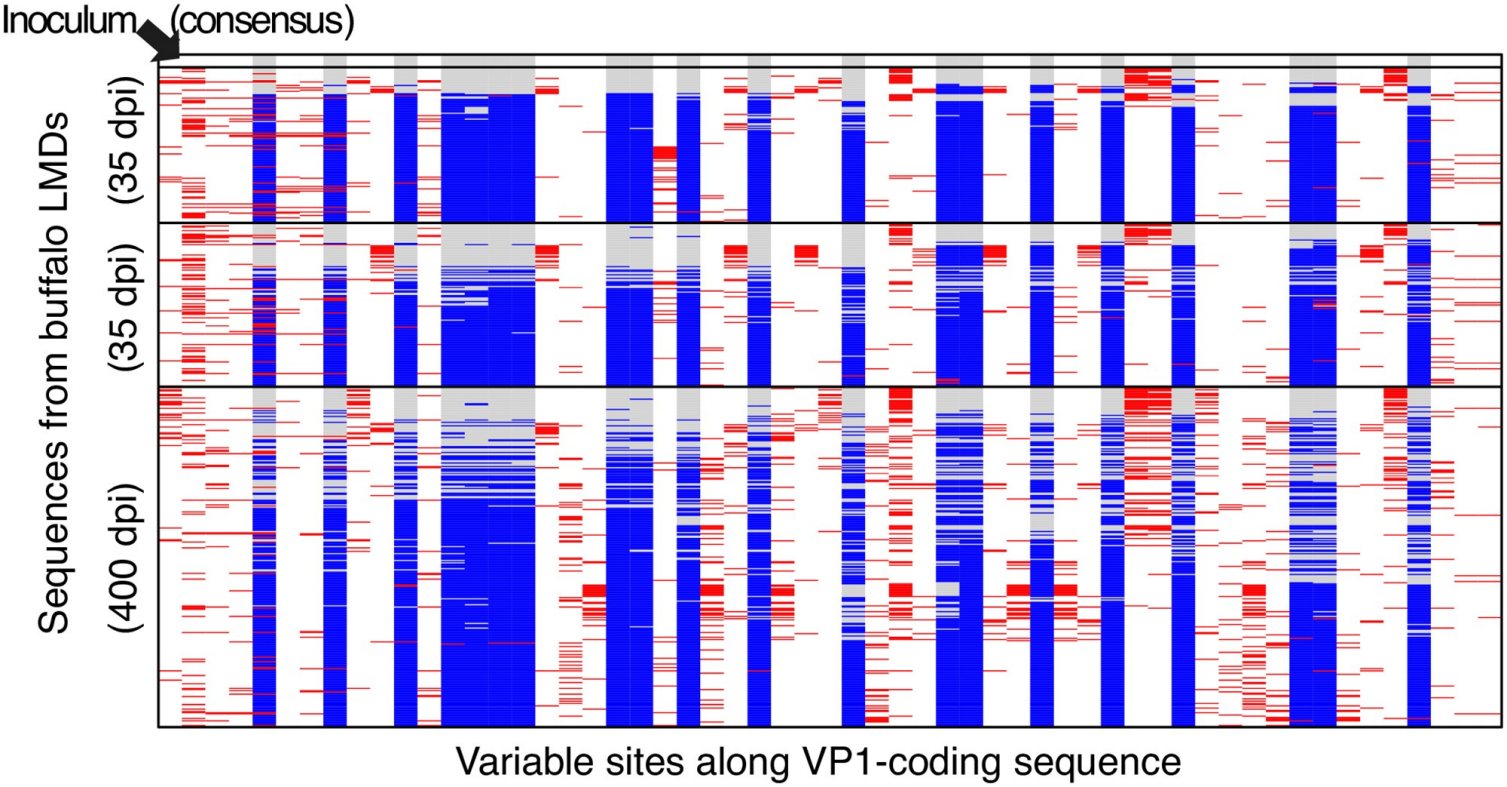
Illustration of the genetic structure of of the VP1-coding sequences from Laser Micro-Dissections (LMDs) of buffalo tissues. Each row represents the genetic content of a sequence and each column represents a SNV. The alleles are illustrated by the following colors: grey (consensus alleles of the inoculum), blue (alleles of the minor sub-population in the inoculum), white (alleles in common between both sub-populations) and red (new mutations with respect to the consensus sequences of the two sub-populations). Sequences are sorted by animal, then by divergence from the consensus sequence of the inoculum (shown at the top); the two sub-populations correspond therefore to the upper (grey-dominated) and lower (blue-dominated) sequences for each animal.

For the buffaloes, samples from Laser Micro-Dissections (LMDs) from several oropharingeal tissues (dorsal soft palate, palatine and pharingeal tonsils) were obtained at day 35 post-infection from two animals (denoted here as buffaloes “19” and “X4”) and day 400 from one animal (buffalo “44”). Sequence from the VP1-coding region were obtained by cloning and Sanger sequencing, resulting in 569 Sanger sequences passing quality controls. The genetic content of these sequences is illustrated in Fig 3.

A sub-population structure similar to the one detected in the inoculum was also found in viral VP1-coding sequences from LMDs of tissues from these three infected buffaloes, demonstrating that co-infection occurred in this experiment. The genetic structure of the swarms is illustrated in Fig 3. These sub-populations show little differentiation between tissues, hence we consider all sequences from the same animal to be part of a single viral population. A detailed analysis of the diversity of the sub-populations across animals and tissues, their evolution post inoculation and the immune response of the animals can be found in [26].

### Recombination between viral sub-populations

Co-infection of buffalo hosts by different viral sub-populations offers an opportunity to observe within-host recombination. In fact, recombination is assumed to occur whenever two viruses co-infect the same cell [20], but it can only be detected when their sequences are different enough to be clearly separated. This is the case in our experiment. Indeed we observe a large number of recombinants among the Sanger sequences of clones derived from the buffalo tissue micro-dissections (Fig 3). Extensive recombination between sequences belonging to the two initial swarms is also detected in the short reads from the inoculum. These observations cannot be due to sequencing errors or repeated mutations; a contribution from artefactual recombination during sample preparation cannot be excluded, but it cannot fully explain the data (see Methods).

**Fig 4:**
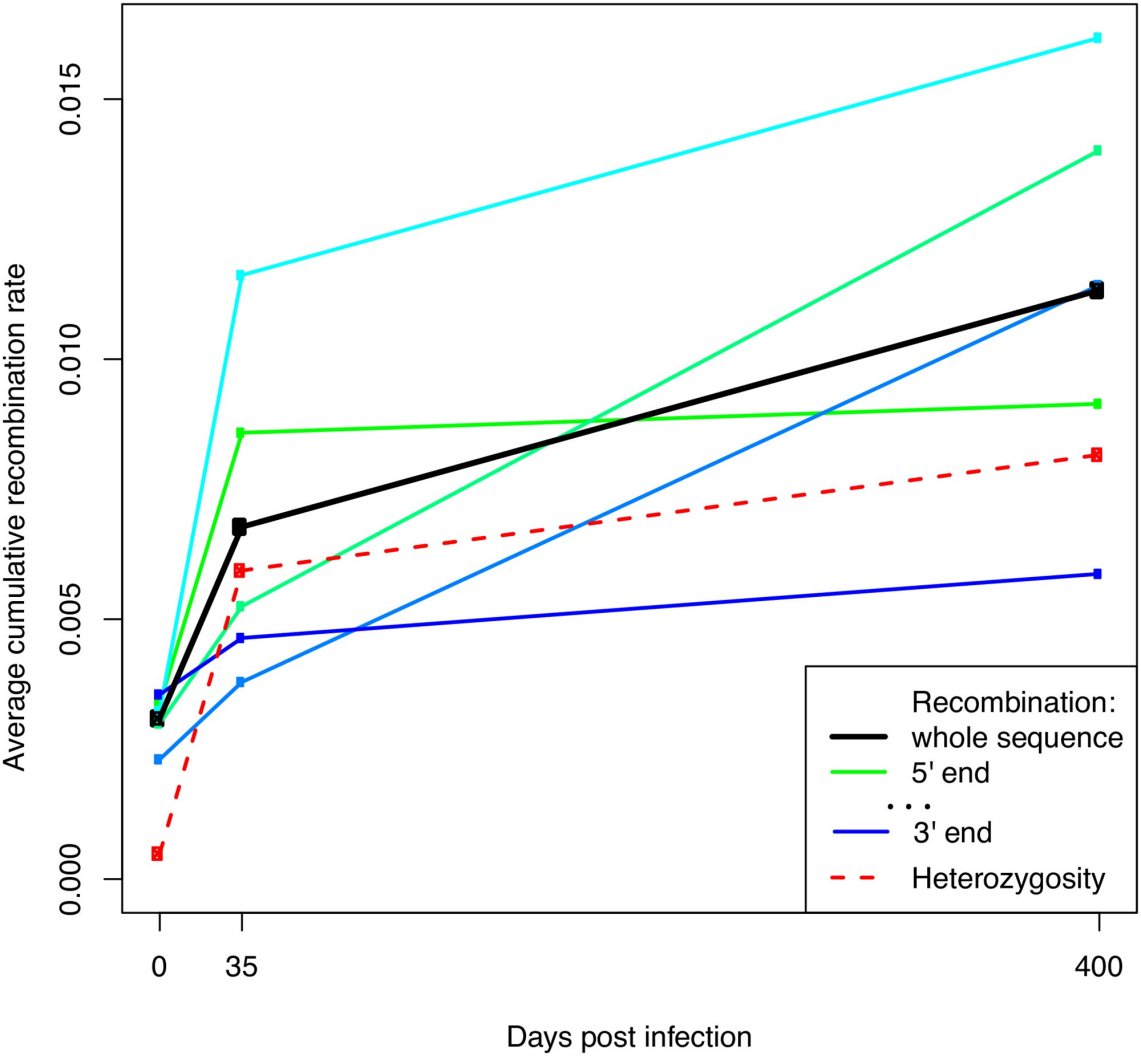
Cumulative recombination rates in VP1-coding sequences from the inoculum and from animals sampled at 35 and 400 dpi. All the rates are defined from the beginning of the experiment to the sampling time of the sequences and are computed using the “local” approach. Recombination rates for the 1st, 2nd, 3rd, 4th and 5th fifth of the the VP1-coding sequence are shown with a green-blue gradient from the 5’ to the 3’ end. The dashed red line shows the heterozygosity per base, computed on the intra-swarm SNVs (i.e. variants unrelated to the main sub-population structure).

The number of recombination events detected in these sequences is surprisingly large: there is at least one recombination event between almost all pairs of Single Nucleotide Variants (SNVs) characterising the two swarms. Furthermore, the fraction of recombinants seems to increase in time post inoculation (Fig 4), suggesting that the mixture of co-infecting swarms has a recent origin and has not reached a stationary equilibrium. These features allow us to apply classical population genetics approaches to this system.

In classical population genetics, recombination can be inferred from *linkage disequilibrium* (LD), a measure of the correspondence between the genotypes of two closely occurring SNVs [27]. In the absence of recombination (and of recurrent mutations), the physical linkage between alleles along the sequence constrains the possible allelic combinations. As an example, for two SNVs originating from a mutation …A…G…→…T…G… in the first site followed by a …A…G…→…A…C… mutation in the second site, the only possible allelic combinations without recombination are {A,G}, {T,G} and {A,C}, i.e. C in the second site would always be found with A in the first, and T in the first site would always be found with G in the second; in addition, {A,C} would tend to appear at lower frequencies. The effect of recombination events between the two SNVs is to reshuffle these allelic combinations; in our example, recombination occurring between the two SNVs would generate sequences with a {T,C} genotype and increase the frequency of the {A,C} genotype. Linkage disequilibrium quantifies the observed extent of reshuffling between genotypes.

There is a direct relation between the strength of LD and the recombination rates. If the sequence between two SNVs recombines at a rate *r* for a time *t* since the formation of the mixture, LD decays over time as an exponential LD ∼ *e*^−*R*^ of the cumulative recombination rate *R* = *r* ⋅ *t* [27]. The decrease in time of the correspondence between alleles at nearby SNVs is illustrated in Fig 1. However, linkage disequilibrium could also be affected by *epistasis*, i.e. fitness-related interactions between genetic variants. In fact, when recombination disrupts favourable combinations of co-evolved variants, recombinants have lower fitness and their number is suppressed by selection. More generally, if different combinations of alleles at multiple loci have different fitness, the frequency of favoured combinations of alleles increases and the synergy between alleles corresponding to these combinations is reinforced. Hence these epistatic interactions often act in opposition to recombination and cause an effective increase in LD [28].

### Absolute recombination rates

Linkage disequilibrium and recombination rates were inferred separately both for inoculum and for the buffalo samples. For the whole capsid-coding region of the inoculum, linkage disequilibrium is computed from all short reads overlapping the pair of polymorphic sites considered. For the VP1-coding sequence of the virus from three buffaloes, two sampled at 35 days post infection (dpi) and one sampled at 400 dpi, linkage disequilibrium is computed from all Sanger sequences. All cumulative recombination rates (i.e. recombination rates integrated over time) are relative to the time of origin of the mixture of sub-populations, which is not known, hence their absolute values do not have any easy interpretation. However, in the absence of biases, their differences provide absolute recombination rates per unit time across the acute and persistent phases of the infection. We can estimate these rates only for VP1, since it is the only genomic region for which multiple time-points (0, 35 and 400 days post infection) are available.

Recombination rates were estimated using LD between pairs of subpopulation-specific SNVs, i.e. variants consistent with the two main sub-populations of the inoculum. Two approaches were used for inference of recombination rates: the “local” approach uses only information from consecutive variants, while the “global” approach uses information from all variants. The “local” approach is therefore more noisy, while the “global” one is more precise but could be more sensitive to biases. The two methods are also affected by epistatic interactions, but at different scales.

The average cumulative recombination rates per base, estimated using the “global” and “local” approaches, are *R*_0_ ≈ 2.6 ⋅ 10^−3^ − 3.0 ⋅ 10^−3^, *R*_35_ ≈ 4 ⋅ 10^−3^ − 7 ⋅ 10^−3^ and *R*_400_ ≈ 8.0 ⋅ 10^−3^ − 11.7 ⋅ 10^−3^ respectively, accumulating in time as illustrated in Fig 4. Hence, the rate per year during the first 35 days post inoculation is *r*_0−35_ ≈ 0.015 − 0.040/site/y, while for later times the rate is *r*_35−400_ ≈ 0.004 − 0.005/site/y. Hence, during the first month post-infection, the average recombination rate is higher by a factor *r*_0−35_/*r*_35−400_ ≈ 3.8 − 8.7. Since the acute phase of the infection lasts about a week [2], the actual rates from the “global” and “local” approach can be estimated as

$$\begin{array}{cc} r_{\text{acute}} & \approx 0.6\cdot 10^{-1}-1.9\cdot 10^{-1}/\text{site}/y\\ r_{\text{persistent}} & \approx 4\cdot 10^{-3}-5\cdot 10^{-3}/\text{site}/y \end{array}$$

i.e. recombination during the acute phase is 15 − 40 times faster than during the persistent phase.

Note that these absolute recombination rates are comparable or even higher than the typical substitution rates for FMDV, which are as high as 10^−2^ mutations per base per year [29, 30] due to the error-prone nature of the RNA polymerase. The rates per site per generation are also of the same order of magnitude as the ones inferred for *in vivo* HIV infections [23].

Viral replication is a prerequisite for recombination [20]. Hence, FMDV keeps replicating in tonsils and other tissues during the carrier phase, albeit more slowly. Under the assumption that the recombination rate is roughly proportional to the replication rate, we could translate the above results in terms of relative replication rates. Using the “local” estimate, under this assumption, viral replication during the carrier state case would proceed about 40 times slower than in the acute phase of the infection. Note that this is a very rough estimate and it could be affected by many sources of bias, including different population structures and selective pressures in the two phases.

### Recombination profile in the capsid-coding region

We now look at the fine-scale structure of recombination rates. The basis for the inference of recombination is the normalised linkage disequilibrium *D*′ between pairs of derived subpopulation-specific variants. The measure *D*′ is defined in the Methods and it takes values +1 or −1 in the absence of recombination, while it is close to 0 for strong recombination. The *D*′ values for pairs of variants in the capsid-coding region are shown in Figure B in S1 File as estimated from high-throughput reads from the inoculum.

**Fig 5:**
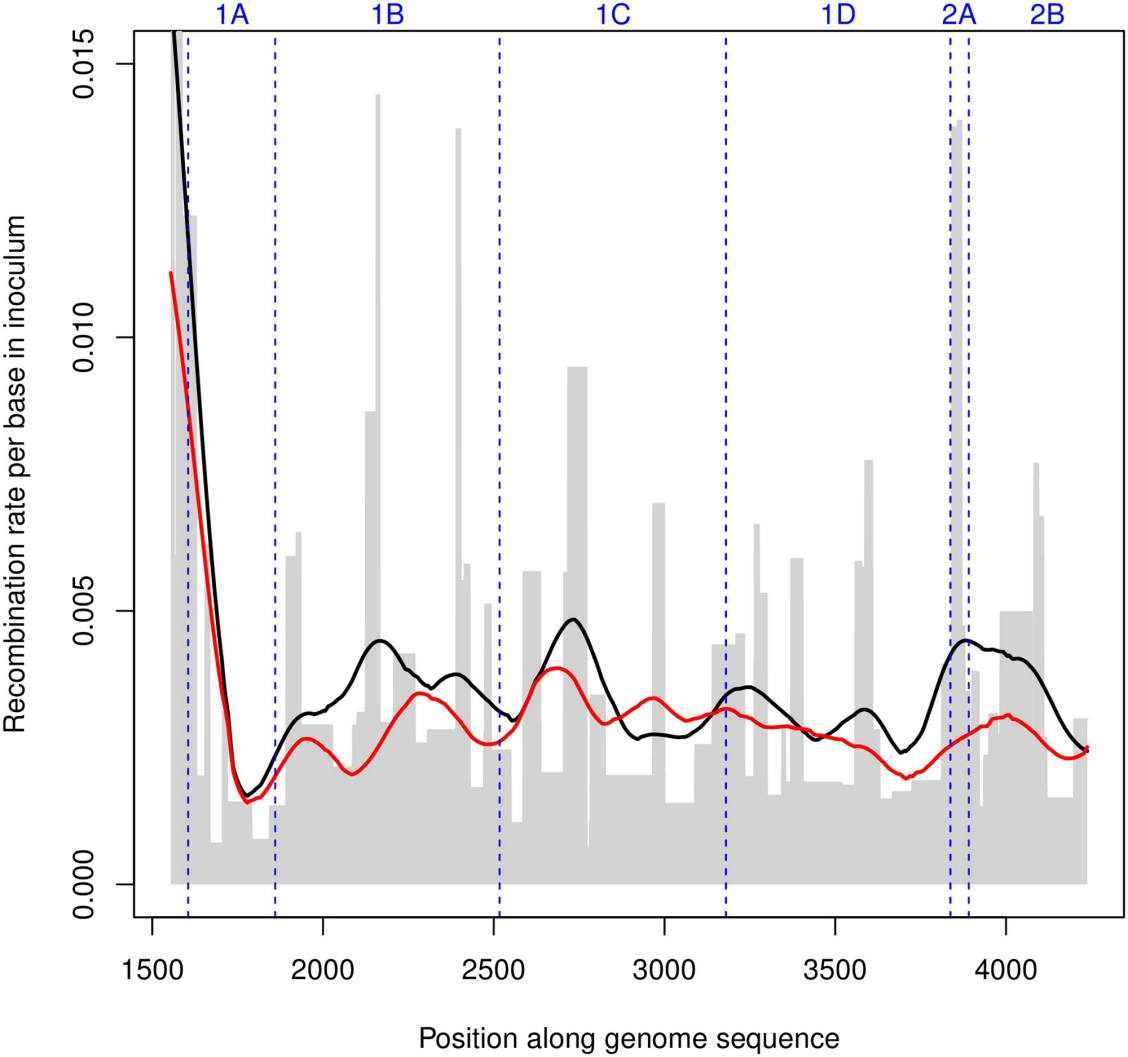
Effective cumulative recombination rate per base for the inoculum (in grey), inferred by the “local” approach from pairs of SNPs covered by at least 10^3^ reads. The lines indicate the average local rates (black) and global rates (red) Gaussian-smoothed over a 150 bases window.

A detailed recombination profile can then be built from *D*′ using the “global” and “local” approaches discussed in the previous section. This recombination map extends almost to the whole sequenced region, i.e. capsid-coding plus flanking regions, and the distance between subpopulation-specific variants (∼ 30 bases on average) determines the resolution of the profile. The final profile is shown in Fig 5.

We observe that recombination rates inferred by the “local” approach for the capsid-coding region peak strongly around the 3’ end of Lpro/5’ end of VP4. They also show a moderate heterogeneity both between and within protein-coding regions, with peaks around the middle of the VP4- and VP3-coding sequence and in the region of 2A-B.

From this recombination map, it is also possible to obtain an estimate of the relative recombination rates with respect to the VP1-coding sequence of the other capsid protein-coding sequences (VP4, VP2, VP3 or 1A-1C) and some non-structural protein-coding ones (2A-2B and small regions at the 5’ end of 2C and at the 3’-end of Lpro). These relative rates are summarised in Table 1.

**Table 1:** Relative intra-host recombination rates inferred among different parts of the capsid-coding sequence and flanking regions.

Region	*R*/*R*_VP1_, global	*R*/*R*_VP1_, local
Lpro	6.2	8.7
2A	1.12	2.8
2B-2C	1.04	1.17
2A-2C	1.05	1.4
VP4 (1A)	0.93	0.87
VP2 (1B)	1.07	1.27
VP3 (1C)	1.26	1.15
1A-1C	1.12	1.16

Recombination rates in flanking regions of the capsid-coding sequence (Lpro and 2A) are higher than in the capsid-coding sequence itself. Hotspots of recombination in the flanking regions of the capsid-coding sequence have been previously detected in studies based on phylogenetic evidence [18, 19]. These previous analyses inferred levels of phylogenetic recombination that were extremely low for VP1- and capsid-coding sequences and much higher for non-structural protein-coding ones. In partial contrast with these studies, we observe high intra-host recombination rates in the capsid-coding region, while the recombination rate in 2A is larger but still of the same order of magnitude as the capsid rate, and the rates in 2B-2C and in the capsid-coding region are actually similar.

### Mosaic structure in the VP1-coding region

**Fig 6:**
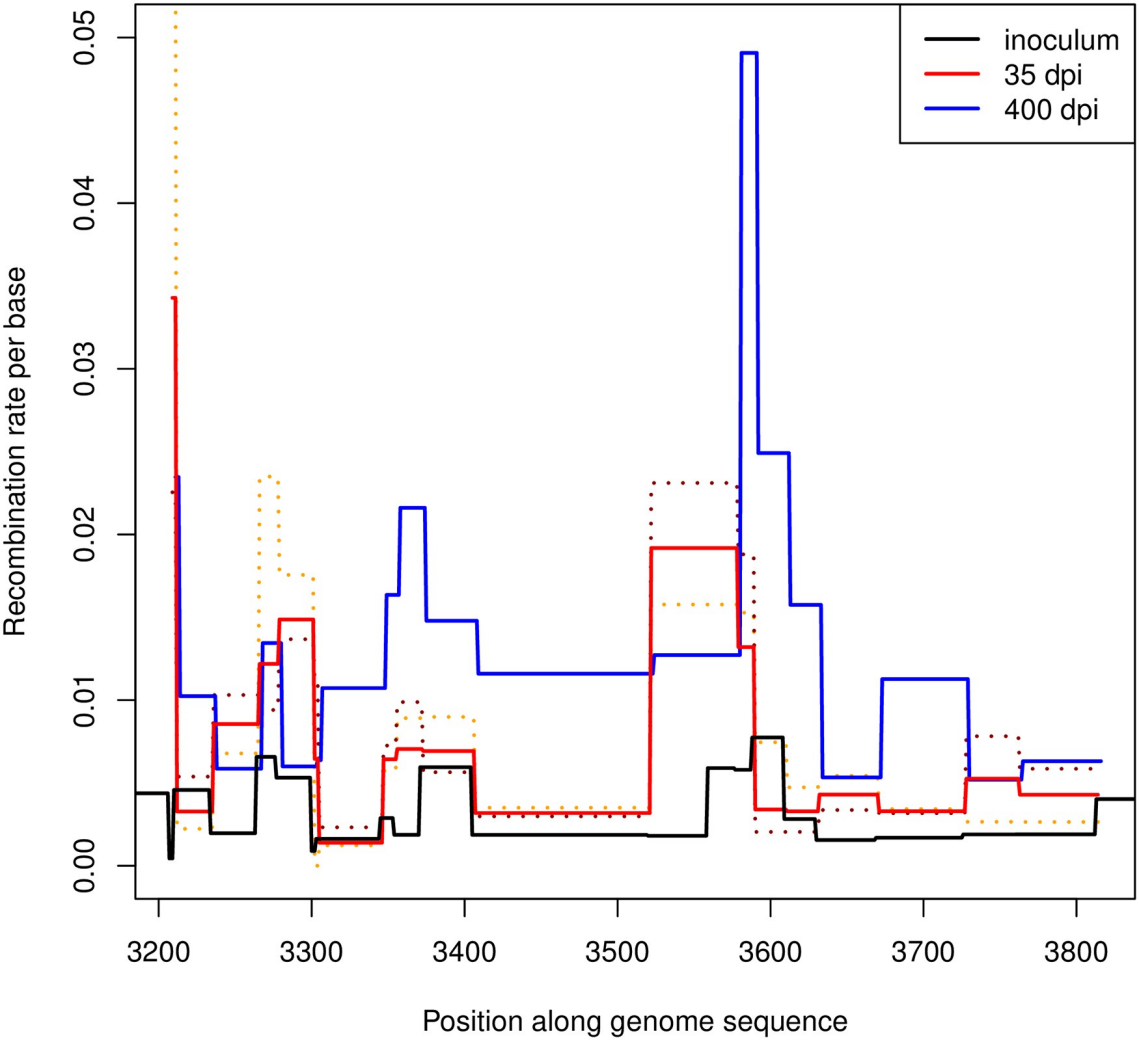
Effective recombination rate per base along the VP1-coding sequence. The cumulative rates are measured from the beginning of the experiment to sampling times: 0 days post inoculation (dpi) i.e. inoculum; 35 dpi; and 400 dpi. To illustrate the heterogeneity in inferred recombination rates between individuals, two separate dashed lines are shown for the two individuals sampled at 35 dpi.

Thanks to our experimental design, recombination profiles for the sequence coding for VP1 (1D) can be reconstructed from different individuals and timepoints: the inoculum, two animals sampled at 35 dpi and an animal sampled at 400 dpi. It is therefore interesting to compare the different profiles. The absolute recombination rates inferred from the “local” approach are shown in Fig 6. On the top of a trend of increasing recombination rates with time (already clear in Fig 4), we observe some heterogeneity in the recombination rates along the protein-coding sequence, with several peaks found in similar locations across different individuals.

**Fig 7:**
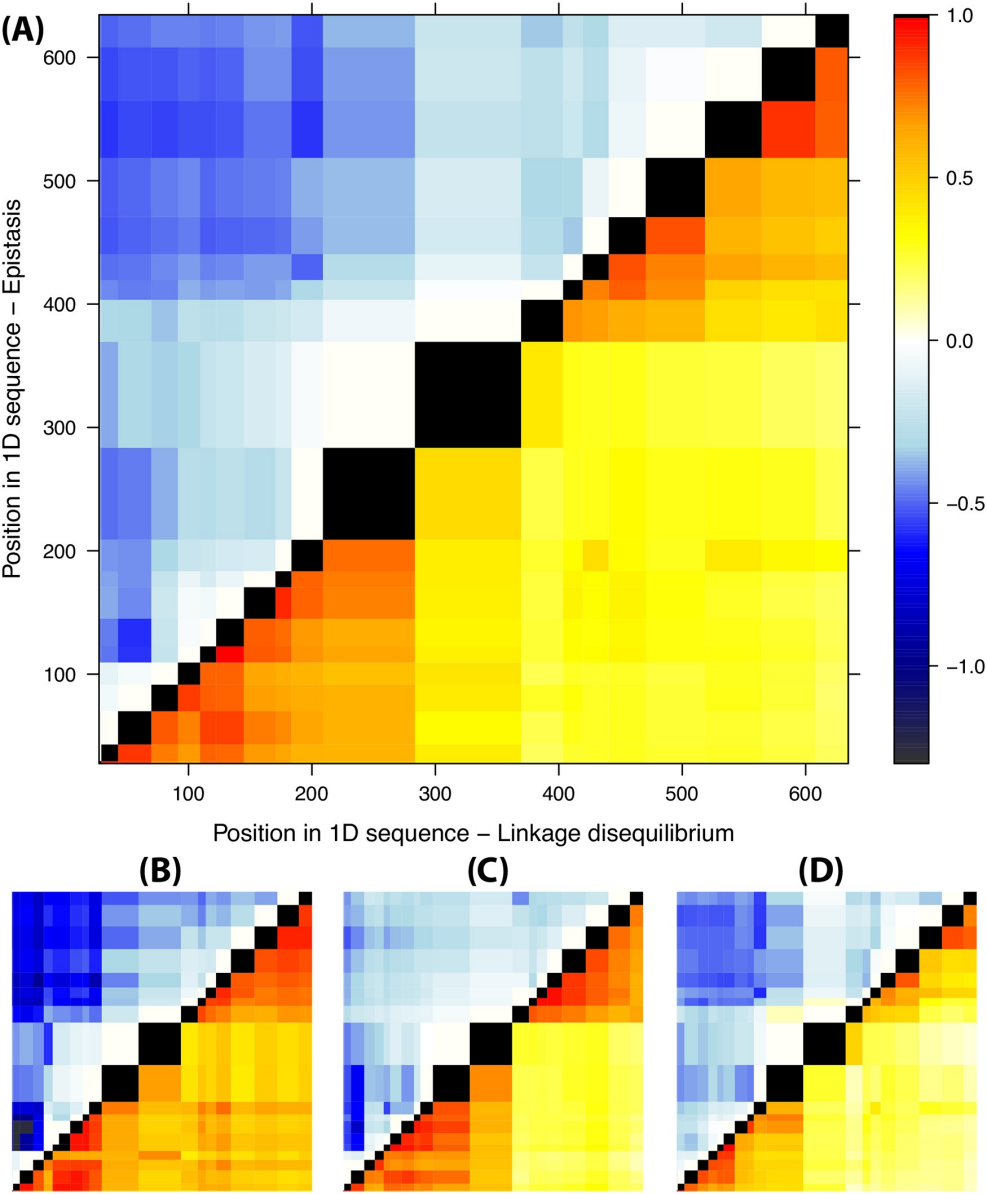
LD and epistasis across sequences from all animals (A), the two individuals sampled at 35 dpi (B,C) and the individual sampled at 400 dpi (D). Lower triangles: map of pairwise LD between variants in VP1, estimated as |*D*′| (stronger LD shown in red). Upper triangles: signatures of epistasis as detected by the suppression of recombination $R-R_{\text{predicted}}=\text{log}(D_{\text{predicted}}^{\prime }/D^{\prime })$ (stronger suppression shown in blue).

The complete VP1-coding sequences from micro-dissections of buffalo tissues reveal a richer structure created by the interplay of recombination and epistasis. In fact, these sequences contain information about the linkage disequilibrium of most pairs of variants within VP1, as they have been obtained by Sanger sequencing. The corresponding LD maps for subpopulation-specific SNVs are shown in Figures 7A–7D (lower triangles). The LD patterns show two regions (or “blocks”) with strong internal linkage |*D*′| ≳ 0.5, i.e. reduced recombination within each block. They roughly correspond to the first 200 and last 250 bases of the VP1-coding sequence. These blocks correspond to the red-orange triangles in the lower half of Fig 7A, and their pattern is broadly consistent across different individuals and times (Fig 7B–7D). This suggests that the mosaic structure observed in the non-structural part of FMDV genomes [19] is not restricted to non-structural proteins, but actually extends to the capsid-coding region. Recombination interacts with other forces within the host to maintain a modular structure with at least two different linked genetic blocks inside the VP1 protein-coding sequence. Interestingly, hotspots of recombination located in the middle of the VP1-coding region have been observed in poliovirus as well [31].

### Epistasis in the VP1-coding region

Epistasis is another major force influencing and possibly driving the intra-host dynamics. The LD between variants in the same protein-coding region can be affected by epistatic interactions due to functional constraints, stability or immune pressure. If the original combinations of variants in the swarms are fitter than the recombinants, the recombination rate is effectively reduced by selection [27, 28]. The effects of recombination rate and epistasis cannot be separated for pairs of consecutive variants, since it corresponds to the scale of the finest resolution of LD, and the only information available at this scale is a single measure of *D*′. However, for distant pairs of variants, it is possible to detect footprints of epistatic interactions from excess of LD with respect to the naive value $D_{\text{predicted}}^{\prime }=e^{-R_{\text{predicted}}}$ estimated from the “local” approach to recombination rates. We use the suppression of recombination *R* − *R*_predicted_ as measure of the impact of epistasis on recombination.

As expected, intra-protein epistatic interactions shape the LD structure of the VP1-coding region. In fact, the suppression of recombination in Fig 7A–7D (upper triangles) hints at the presence of epistatic interactions inside both genomic blocks in the VP1-coding region. These interactions could contribute to its modular structure [32]. Interestingly, strong signatures of epistasis are found between the two blocks as well. This indicates that even if recombination tends to decouple the two blocks, linkage equilibrium is prevented by epistatic interactions between the blocks, which suppress replication and infectivity of recombinant sequences.

### Pairwise epistatic interactions

Given the large number of interacting variants, it is difficult to disentangle the strength of each pairwise interaction from the cooperative epistatic effects of all other linked variants; their cumulative effect could lead to “genotype selection”, i.e. locking variants into haplotypes containing only the most favourable combinations [32].

In order to disentagle the effect of pairwise interactions, we modify the local prediction of recombination rates to a nonlocal heuristic pairwise prediction *R*_2,predicted_ that accounts for the suppression due to the most strongly linked chain of variants between each pair. While the effective suppression of recombination *R* − *R*_2,predicted_ could be used as a signature of pairwise epistatic effects, we can use a population genetics approach to infer the cumulative strength of selection against recombinants *s*′ based on an explicit model of evolution with pairwise epistatic interactions (see Supplementary Section S8). Selection coefficients leads to qualitatively similar results as the suppression of recombination, but also provide us with a well-grounded estimate of the strength of selection, even if entangled with the time since formation of the sub-populations.

With this finer approach, the observed strength of selection *s*′ (derived from *R* versus the heuristi prediction *R*_2,predicted_) confirms the presence of interactions both within and between the two genomic blocks in VP1 (Figure J in S1 File). While the suppression of recombination is much stronger between blocks, the inferred pairwise interactions that connect the blocks are few and differ between animals (Figures H-M in S1 File). This suggests that the linkage between the blocks does not originate from a single pairwise interaction, but from the cumulative strength of cooperative interactions between multiple variants, possibly through selection at the haplotypic level (“genotype selection” in the definition of [32]) and/or higher-order epistasis with synergistic patterns of interaction.

**Fig 8:**
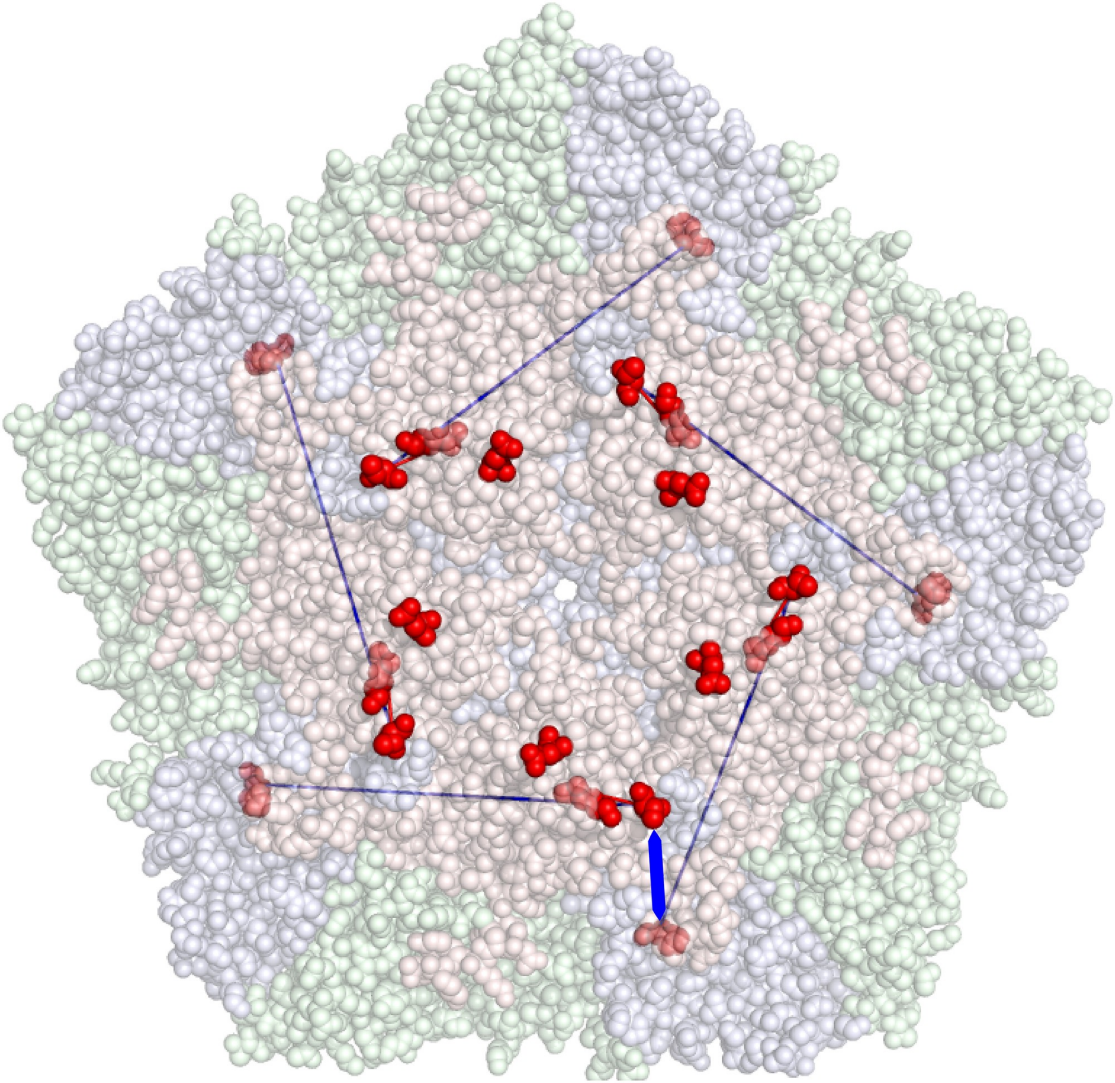
Localisation of the aminoacids corresponding to the four subpopulation-specific non-synonymous variants involved in epistatic interactions, projected on the capsid structure of SAT1. The red, green and blue components of the capsid correspond to VP1, VP2 and VP3 respectively. Exposed aminoacids corresponding to the variants are shown in dark red. Two epistatic interactions are illustrated, a strong one between H18Y and A99T (in blue) and a weaker one between K49R and A99T (in red). Note that the first interaction could also occur between residues H18Y and A99T from two different VP1 proteins (thick blue arrow), which are physically much closer in the capsid structure.

Within each block, we find strong pairwise epistatic interactions with coefficients up to *s*′ ∼ 2. In general, we observe that the strength of interactions between non-synonymous variants is higher than between synonymous ones (Figure P in S1 File). In fact, the strength of epistatic interactions between pairs of non-synonymous variants is significantly higher than between other pairs at similar distances (*p* = 0.006): this proves that intra-host epistatic selection pressures acts at the protein level as well. Aminoacid interactions are illustrated in Fig 8. The strongest aminoacid interaction involves the variants H18Y and A99T in VP1 and is likely to be related to the stability of the capsid structure. On the other hand, there is no clear signature of selection on stability of the RNA secondary structure (Supplementary Section S10), although this could be due to the fact that the subpopulation-specific variants found in this experiment are quasi-neutral with respect to RNA structure.

In order to check the robustness of our heuristic approach to detect pairwise epistatic interactions, we develop a completely independent approach for the inference of direct pairwise interactions. This second approach is closely related to Direct Coupling Analysis [33], which is a class of successful methods able to extract direct interactions between pairs of protein residues from the pairwise correlations of the genetic variability at these positions across multiple species. In Supplementary Section S9, we develop an approximation to the DCA based on *D*′ and inspired by [34]. Our DCA approach is able to disentangle direct interactions not only from the effect of indirect interactions, but of physical linkage as well. While the DCA approach detects many more putative interactions than our heuristic approach, the strength of the variants detected by both approaches is very well correlated (Figure P in S1 File), despite the widely different nature of the two approaches. This confirms the robustness of our heuristic approach to infer the strengths of pairwise selection.

Overall, these results imply that epistatic interactions are widespread inside the VP1-coding region. Intra-host selection acts both at the RNA and protein level and epistatic interactions exist at both levels. In our experiment, these interactions reduce by half the rate of recombination between blocks. Pairwise interactions within blocks reach a strength of selection up to *s* ∼ 0.1 between pairs of variants.

## Discussion

### Intra-host recombination map for the capsid sequence

In this paper we provide the first inference of the recombination maps for intra-host FMDV evolution. Recombination maps were inferred both for the whole capsid-coding region and for VP1. Our results are limited to infections of a single serotype (SAT1) in a single host (buffalo). In principle, the details of the recombination map might differ between serotypes. The absolute rates and the amount of epistasis are also expected to depend on the within-host infection dynamics and the immune response of the host, hence they could vary as well when considering infections in cattle or other species. However, we do not expect the qualitative picture to differ essentially for other host species and serotypes.

We were also able to detect intra-host epistasis on the scale of the VP1 sequence. We discovered that epistatic interactions are widespread among the variants appearing in this study. The main effect of these epistatic interactions is to maintain the genetic linkage between two large genomic blocks located within VP1. Some of these interactions occur between aminoacids and play a role in the stability of the capsid.

It is difficult to exclude a contribution from recombination during passage in cell culture or sample preparation. Passage before sequencing could definitely contribute to the number of recombinants. However, it is unlikely to have a major effect on the total number of recombinants. In fact, the inoculum has been passaged several times before sequencing, hence it should have the largest contribution from recombination in culture, but its cumulative recombination rate is much lower than the ones from buffaloes (see Fig 4). Therefore, most recombination events observed in this study can be most likely attributed to replication in buffaloes. Recombination during PCR amplification could also be an issue, but it is unlikely to be the dominant contribution in this experiment. Note that the recombination profiles during library preparation (and most likely also during passage) could resemble closely the profiles in vivo, as shown in [31] for polioviruses.

The inferred recombination rates are related to the amount of co-infections of the same cells in the host, hence they depend intrinsically on within-host dynamics [20]. More generally, recombination rates are influenced by the fact that they are computed based on visible recombination events only, as discussed in Supplementary Section S7. This is unavoidable in all studies of viral recombination in vivo. In fact, it is better to consider our results as “effective recombination rates” which already take into account the effect of within-host evolution and infection dynamics. Hence, they already include the impact of within-host genetic structure, selection against recombinants and generation of non-viable recombinants.

Recombination rates are also related to replication rates. In this experiment we observe an increase in time both in cumulative recombination rates and in haplotypic diversity during persistent FMDV infections in buffaloes. This suggests that FMDV replicates in the carrier phase as well, albeit more slowly than during the acute phase of the infection.

Inferred recombination rates depend on epistasis as well. This is unavoidable even when considering high-resolution maps such as ours, since we have no way to account for the effect of local epistatic interactions between close mutations. This also means that “local” estimates, which are affected only by local epistasis, should be more reliable than “global” estimates, which are more likely to systematically underestimate the real rates due to the additional effect of longer-range epistasis and the cumulative effect of cooperative interactions among multiple linked variants. In fact, such underestimation appears clearly even in Fig 5. This is also the reason why we have shown only “local” estimates for most results.

A likely explanation for the suppression of recombination in the capsid-coding region is the presence of widespread epistatic interactions acting at the level of the capsid structure and possibly the RNA structure as well. In fact, we found two such pairwise aminoacid interactions in this work, with a strength of selection up to *s* ∼ 0.1 per generation. Pairwise interactions were inferred from two different approaches and represent therefore a strong finding of this paper. Epistasis among many variants was also indirectly detected from the suppression of recombination between the two genomic linkage blocks in VP1. The overall selection against recombinants caused by epistatic interactions between these blocks can be estimated as *s* ∼ 0.1 (Supplementary Section S11), illustrating how the disruption of co-evolved combinations of alleles carries a high intra-host fitness cost for the virus.

### Consequences for the evolution of the FMDV capsid

The high recombination rates in structural proteins between genetically close lineages represent an important finding of this work. In fact, it is natural to assume that recombination between genetically closer sequences will be even higher. This has potentially relevant implications for the genetic diversity in quasi-species. In fact, mutation and recombination play different roles in generating genetic diversity, and their balance can affect the fate of the quasi-species, as recently suggested in [35]. Mutations have a direct effect on the diversity of the swarm by generating new nucleotide variants, but a high mutation rate also adds a significant load to the population, as most of these polymorphisms are deleterious and tend to reduce the overall fitness of the quasi-species [9]. On the other hand, recombination plays an indirect role, by generating different combinations of existing nucleotide variants (see Section S11). This increases the haplotypic diversity of the swarm while unlinking the fate of potentially advantageous and deleterious mutations, increasing the chances of compensatory combinations of mutations, and reducing the probability of fixation of deleterious mutations [35, 36]. All these effects alleviate the mutational load. Hence, although the actual role of recombination in RNA viruses is still unclear [37], high intra-host recombination rates could be beneficial for FMDV quasi-species [20].

Recombination events could even generate new genetic diversity at a phylogenetic level in sequences coding for capsid proteins, provided that they are not suppressed by lineage competition or epistatic selection against recombinants (see discussion in Supplementary Section S12). A possible mechanism could be the exchange of short sequence fragments between different viral strains, caused by multiple intra-host recombination events during co-infections. Intriguingly, we find suggestive evidence for this mechanisms among FMDV sequences from tonsil swabs (Section S11). Further studies are needed to understand which phenomena suppress capsid recombination on broad epidemiological scales and which viruses with a recombinant capsid-coding sequence could represent an epidemiological risk.

### Conclusions

In this paper we present the first inference of within-host recombination rates for structural proteins of FMDV. This study is possible thanks to a co-infection of two SAT1 viruses, creating two co-occurrent subpopulations with a small sequence divergence of about 3% inside the buffalo hosts. The recombination rates during the acute and persistent phases of the infection are about *r*_acute_ ∼ 0.2/site/y and *r*_persistent_ ∼ 0.005/site/y. This shows that intra-host recombination rates are high and even higher than the substitution rate. It also suggests that the virus keeps replicating in tonsils and other tissues during the carrier phase, although at a much slower rate than in the acute phase of the infection. We provide high-resolution maps of recombination at the scale of the capsid-coding and flanking regions, showing that recombination is a pervasive phenomenon in the FMDV genome. We also discover a modular structure in the VP1-coding region, formed by two strongly linked genomic blocks. Linkage within and between blocks is maintained by widespread epistatic interactions between beneficial combinations of co-evolved variants as well. These selective pressures act both at the protein and the RNA level. The strength of these epistatic selection coefficients is up to *s* ∼ 0.1 per replication.

Our results suggest that recombination and epistasis play an important and unappreciated role in the evolution of FMDV. Within-host recombination reduces the mutational load and is likely to give a strong contribution to the creation of intra-host haplotypic diversity in FMDV swarms/quasi-species. During co-infections, it could also transfer genetic diversity from one strain to the other via recombination-mediated exchange of short RNA fragments, hence contributing to the between-host evolution of FMDV sequences and the genetic diversity of the virus at broader scales. However, pervasive epistatic interactions between co-evolved variants would prevent the spread of viruses with recombinant capsid sequences. These interactions might be the key factor for “speciation” of FMDV serotypes at the capsid level.

## Materials and methods

### Experimental setup, sequencing and analysis

African buffaloes were co-infected with a mixed inoculum containing FMDV SAT1/KNP/196/91 (Accession Number KR108948) as well as SAT2 and SAT3 strains during a challenge experiment performed in the Kruger National Park (South Africa). More details of the experiment are explained in [2]. Viruses used for the inoculum originated from African buffalo and were amplified in cell culture using porcine cells 369 PK15 (one passage) and IB-RS-2 cells (5 passages), then libraries were sequenced on a MiSeq (Illumina) obtaining paired end reads of approximately 150 bp each. More details on the sequencing approach can be found in the Supplementary Section S1 and in [26].

A reference sequence for the inoculum was assembled using a sensitive in-house pipeline (Ribeca et al, in preparation) based on SPAdes [38] and additional bespoke software (see Supplementary Section S2). Reads were aligned to this sequence using the GEM mapper [39] version 3. For the inoculum, reads were mapped to the assembly with a mean read depth of about 30000. All genome positions given in the text are relative to the sequence of SAT1/RV/11/37, which is the prototype of SAT1 viruses. The sequenced region comprises the 3’ end of Lpro, the capsid-coding region as well as 2A, 2B and the 5’ end of 2C, and it aligns to the genomic region SAT1:1562-4579.

Samples from Laser Micro-Dissections from three oropharingeal tissues (dorsal soft palate, palatine and pharingeal tonsils) were obtained from buffaloes “19” and “X4” at 35 dpi and from buffalo “44” at 400 dpi. The VP1-coding region of 56 of these samples was amplified using VP1-specific primers, cloned and sequenced on an ABI PRISM 3730 analyser (Applied Biosystems), resulting in 569 SAT1 sequences after strict quality filtering. The length of these sequences is 674 nucleotides. Multiple alignment of the assembled sequence of the inoculum with the sequences obtained by Sanger technology was performed by Clustal Omega [40].

### Population structure

SNV variants in the inoculum were called by a in-house pipeline using an approximation of the Bayesian calling algorithm in Snape-pooled [41] suitable for high coverage. We considered biallelic variants only and selected the SNVs with *p*-value < 0.05. The sequence of SAT1/KNP/196/91 was used to infer the ancestral allele for each SNV. The derived frequency distribution in the inoculum is clearly bimodal with a gap between 0.15 and 0.20 (Fig 2A). That makes it easy to separate all SNVs in two classes: common nucleotide variants (0.20 < *f* < 0.55) and the low-frequency variants (*f* < 0.15). The first class contains the variants that differentiate between the subpopulations, while the second is the internal variability of the two swarms.

We estimated the linkage disequilibrium (LD) by the normalised measure *D*′ among all pairs of common variants covered by at least 10^4^ reads. This measure is defined as *D*′ = *D*/*D*_max_ if *D* > 0 and *D*′ = *D*/|*D*_min_| if *D* < 0, where *D* is the classical linkage disequilibrium *D* = *f*(*A*_1_*A*_2_) − *f*(*A*_1_)*f*(*A*_2_) for two SNVs with ancestral alleles *A*_1_ and *A*_2_, while *D*_max_ and *D*_min_ are its maximum and minimum possible value given the frequencies of the variants at the two SNVs [27].

The local haplotype structure of the population, with two haplotypes containing ancestral and derived SNV alleles, is clearly illustrated by the concentration of allele frequencies around a value of 0.4 (Fig 2B) and by the high LD between consecutive common variants. In fact, almost all values of |*D*′| are between 0.75 and 1 (Fig 2B). Note that a few mutations have *D*′ ≈ −1, suggesting an erroneous inference of their ancestral state.

We also extracted all nucleotide variants among Sanger sequences and filtered out unreliable SNVs by fitting an empirical model of sequencing errors to our data (see Supplementary Section S3). After filtering, the VP1-coding sequences from micro-dissections of buffalo tissues show a similar pattern of genetic diversity and LD, already apparent in Figs 3 and 7, although with a larger fraction of intra-swarm variants.

### Evidence of within-host recombination

**Fig 9:**
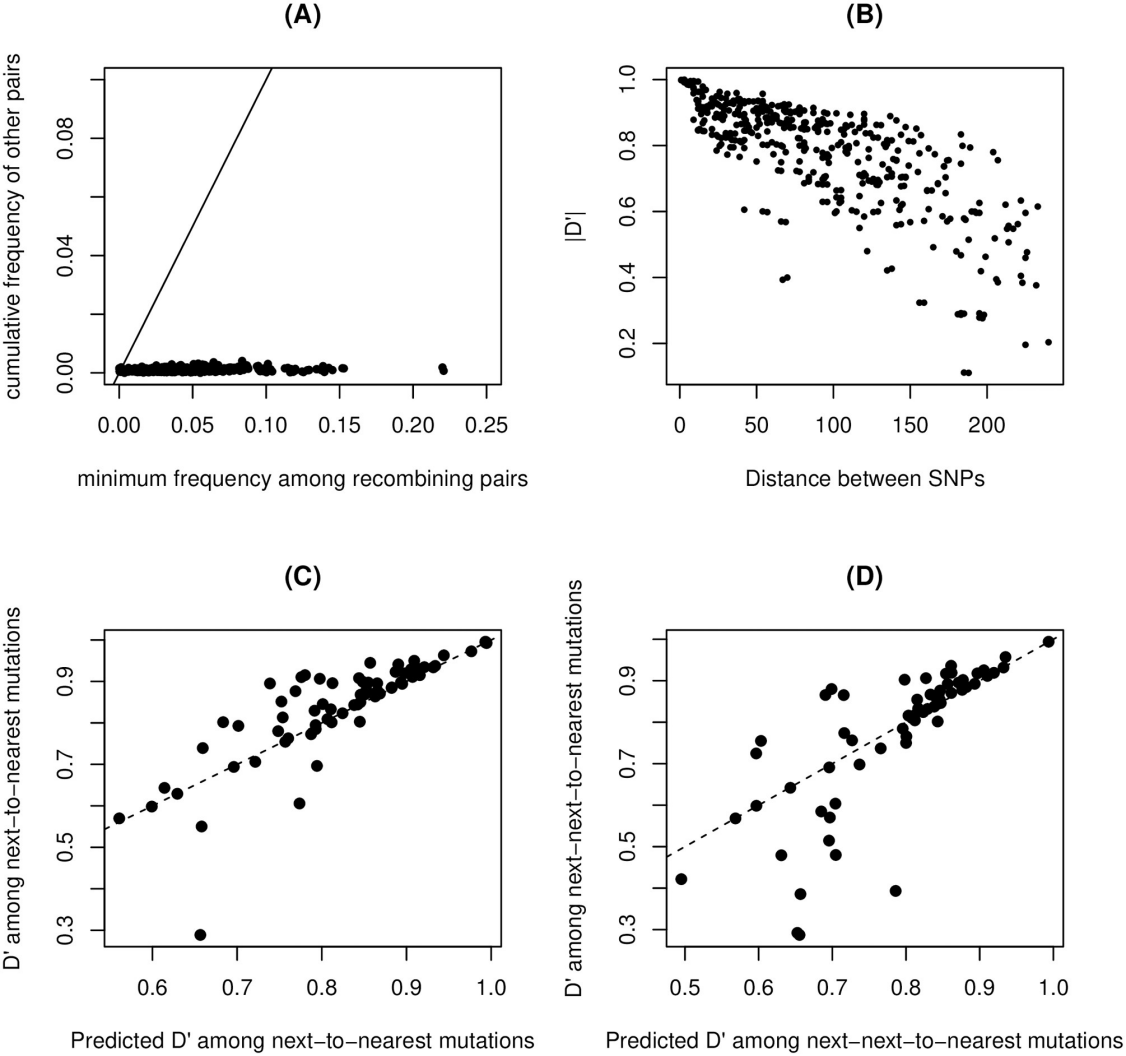
A: Frequency of the least frequent pair of recombinant alleles *f*_*r*,min_ versus the cumulative frequency *f*_*o*_ of all other pairs. For example, if the two main alleles are (C,T) in the first site and (A,G) in the second, then the recombinants are CA, TG, CG, TA; if *f*_CA_ = 0.5, *f*_TG_ = 0.2, *f*_CG_ = 0.1, *f*_TA_ = 0.15, *f*_CC_ = 0.03, *f*_TC_ = 0.02, then *f*_*r*,min_ = 0.1 and *f*_*o*_ = 0.05. Data points are shown for all pairs of polymorphic alleles of frequency > 0.25 covered by at least 10^4^ reads. The black line corresponds to the case of equal frequency. B: Decay of LD measure |*D*′| with distance between SNVs. Data points are shown for all pairs of polymorphic alleles of frequency > 0.25 covered by at least 10^4^ reads. C: *D*′ among next-to-nearest SNVs versus the predicted value from $D_{ik}^{\prime }=D_{ij}^{\prime }D_{jk}^{\prime }$. The dashed line corresponds to equality between predicted and estimated value. D: Same but for next-next-to-nearest SNVs.

The main evidence of recombination in the capsid region comes from the LD data from the inoculum in Figures B and E in S1 File. There are clearly many pairs of mutations with low linkage disequilibrium (−1 ≪ *D*′ ≪ 1), which is a characteristic signature of recombination. Low values of LD can be due to recombination or other spurious factors: (i) sequencing errors; (ii) chimeric reads or similar artefacts of sequencing protocols, generated by recombination during sample preparation or sequence amplification; (iii) multiple mutations/backmutations in mutation hotspots. However, none of these factors except recombination can fully explain the patterns in our data:

- Sequencing errors cannot explain the fact that most putative recombinants contain precisely the same alleles as the two major quasi-species, unless the error rates at each SNV location are extremely skewed towards the same pair of alleles already present. Such an extreme bias seems highly unlikely. Even assuming a moderate bias in error rates, if LD would be caused by sequencing errors, they would be expected to contribute a number of other variants with frequencies similar to the minimum frequency among the four possible pairs of alleles. Instead, Fig 9A shows that the actual contribution of sequencing errors is negligible compared to the predicted one needed to explain our data. A similar argument suggests that mutation hotspots represent an unlikely explanation for the data unless all mutations in these hotspots show a very strong bias towards the two alleles observed at these sites (i.e. most mutations at both sides are back-and-forth mutations between the two alleles).
- Mutation hotspots and sequencing errors cannot explain a pattern of decay of LD with distance along the genome, since they would occur at each site independently, no matter the location. But a clear decay of LD with distance is precisely what is observed in the data (see Fig 9B for the inoculum, and Fig 7 for sequences from buffalo tissues), ruling out these explanations. More quantitatively, recombination implies that the mean LD between positions *i* and *j* satisfies $|D_{ij}^{\prime }|∼e^{-R_{ij}}$ where $R_{ij}=\sum_{x=i}^{j}R(x)$ in terms of the cumulative recombination rate per base *R*(*x*). This implies the approximate prediction $\left|D_{ik}^{\prime }|\approx |D_{ij}^{\prime }D_{jk}^{\prime }\right|$ for *i* < *j* < *k*. The results and the predictions for next-to-nearest and next-next-to-nearest SNVs are shown in Fig 9C and 9D respectively. As for the decay of LD, these patterns cannot be replicated by sequencing errors or by back-and-forth mutations.
- The decay of LD is found both in Sanger- and high-throughput-sequenced samples. It is extremely unlikely that these different protocols would generate chimeric reads with similar profiles. Furthermore, chimeric reads and sequencing errors could not cause the decrease in LD (i.e. the increase in cumulative recombination) over time in Fig 4, since they do not depend on time points but on protocols only.
- Finally, for the buffalo culled at 400 dpi, we are able to compare samples from micro-dissections and from a tonsil swab. Deep sequencing of the tonsil swab shows little internal variability. At the consensus level, the sequence of the swab is a complex recombinant of the two initial swarms, with the sequence of 1A-1B (VP4-2) mostly derived from the major sub-population in the inoculum and 1C-1D (VP3-1) from the minor one. This consensus-level evidence cannot be fully attributed to chimeric sequences (although it cannot exclude that recombination during sample preparation contribute to the observed recombination) and rules out sequencing errors as well. Tonsil swabs and probangs from other animals also present similar features, although with a reduced contribution of the major n of the inoculum.

### Linkage disequilibrium and recombination rates

The high levels of positive LD between all the subpopulation-specific SNVs supports a recent origin for the mixture of swarms. Based on this, we can infer the cumulative rate of recombination since the origin of the sub-populations using the classical equations for the decay of LD with time: *D* = *D*_0_*e*^−*r*⋅*t*^, where *r* is the recombination rate per generation and *t* is the time in number of viral generations [27]. The cumulative recombination rate *R* = *r* ⋅ *t* for the genomic region between two variants can then be inferred as

$$\begin{array}{c} \hat{R}=-\ln\left(D^{\prime }\right). \end{array}$$ (1)

We apply two different statistical approaches to infer the recombination rate for each variant-free interval. The first (“local” approach) is based on the above estimate (1) for consecutive variants only. The second (“global” approach) is given by the weighted least squares estimate from all variants, described in Supplementary Section S5; its disadvantage is that is inherently more affected by epistatic interactions.

Data from Sanger sequencing of viruses from micro-dissections reveals only weak differentiation between tissues from the same animal. The average estimate of $\hat{R}$ across tissues and the joint estimate from all tissues differ by less than 10%, hence we neglect differences across tissues and compute *D*′ from the pooled set of all sequences from a given animal.

### Epistasis

To detect signatures of epistasis, the cumulative recombination rate between the *i*th and *j*th variant is inferred according to Eq (1). The local prediction for the rate is the sum of all rates for the sequence between the *i*th and *j*th variant $R_{\text{predicted},ij}=\sum_{k=i}^{j-1}R_{k,k+1}$.

To detect direct pairwise epistatic interactions, we also implement a heuristic prediction defined as $R_{2,\text{predicted},ij}={\text{min}}_{\left\{k_{1}\ldots k_{s}\right\}}(R_{i,k_{1}}+R_{k_{1},k_{2}}+\ldots +R_{k_{s-1},k_{s}}+R_{k_{s},j})$. This prediction takes into account the suppression of recombination caused by the the most strongly linked chain of variants linking the *i*th and *j*th variant, hence *R* versus *R*_2,predicted_ provides a better estimate of the epistatic suppression due to the pairwise interaction between the *i*th and *j*th variant alone.

Epistatic selection coefficients *s* are inferred from the solution of

$$\begin{array}{cc} \frac{4q\left(1-q\right)s^{\prime }}{R_{2,\text{predicted}}}\left(1-e^{-R}\right)= & 1+\frac{s^{\prime }}{R_{2,\text{predicted}}}-\gamma \coth[\frac{\gamma R_{2,\text{predicted}}}{2}+\frac{1}{2}\text{log}(\frac{1+\frac{s^{\prime }}{R_{2,\text{predicted}}}+\gamma }{1+\frac{s^{\prime }}{R_{2,\text{predicted}}}-\gamma })] \end{array}$$ (2)

$$\begin{array}{cc} \gamma = & \sqrt{{\left(1+s^{\prime }/R_{2,\text{predicted}}\right)}^{2}-8q\left(1-q\right)s^{\prime }/R_{2,\text{predicted}}} \end{array}$$ (3)

where *s*′ = *s* ⋅ *t* and *t* is the time in generation since the beginning of the experiment and *R* is the estimate $\hat{R}$ of the cumulative recombination rate between the mutations (see Supplementary Section S8 for details).

The mathematical details of our approach to Direct Coupling Analysis are developed in detail in Supplementary Section S9. In the Gaussian approximation [34], the strength of direct couplings inferred for non-consecutive variants can be simply extracted from the relation *J*_*ij*_ = (*D*′^−1^)_*ij*_.

## Supporting information

S1 File Supplementary methods and figures. Supplementary Information containing further details on statistical methods, data analysis and evolutionary consequences.
(PDF)
