# Correction: Pervasive within-host recombination and epistasis as major determinants of the molecular evolution of the foot-and-mouth disease virus capsid

**DOI:** 10.1371/journal.ppat.1009050

**Published:** 2020-10-28

**Authors:** Luca Ferretti, Eva Pérez-Martín, Fuquan Zhang, François Maree, Lin-Mari de Klerk-Lorist, Louis van Schalkwykc, Nicholas D. Juleff, Bryan Charleston, Paolo Ribeca

The following information is missing from the Funding statement: Nicholas D. Juleff was funded by Wellcome Trust intermediate-level research fellowship 091726/Z/10/Z.

In the Population structure section of the Materials and Methods, a reference is omitted from the first sentence of the first paragraph.

The correct sentence is: SNV variants in the inoculum were called by SiNPle (Ferretti et al, 2019) using an approximation of the Bayesian calling algorithm in Snape-pooled [41] suitable for high coverage.

The reference is: Ferretti L, Tennakoon C, Silesian A, Freimanis G, Ribeca P. SiNPle: Fast and Sensitive Variant Calling for Deep Sequencing Data. Genes 2019, 10, 561.
