# Supplementary material for: Pervasive within-host recombination and epistasis as major determinants of the molecular evolution of the foot-and-mouth disease virus capsid
Source: PLoS Pathog. 2020 Jan 6;16(1):e1008235. doi: 10.1371/journal.ppat.1008235 (PMC6964909; doi:10.1371/journal.ppat.1008235)
Supplement: S1 File — Supplementary Information containing further details on statistical methods, data analysis and evolutionary consequences. (PDF) [file ppat.1008235.s001.pdf]

Supplementary Information: Pervasive within-host recombination and epistasis as major determinants of the molecular evolution of the Foot-and-Mouth Disease Virus capsid

Luca Ferretti[1,2,*], Eva Pérez-Martín[1], Fuquan Zhang[1],
François Maree[3,4], Lin-Mari de Klerk-Lorist[5], Louis van Schalkwykc[5],
Nicholas D Juleff[1,6], Bryan Charleston[1], Paolo Ribeca[1]

[1]The Pirbright Institute, Ash Road, Woking, Surrey, GU24 0NF, United Kingdom
[2]Current address: Big Data Institute, University of Oxford, United Kingdom
[3]Transboundary Animal Disease Programme, ARC-Onderstepoort Veterinary Institute, Private Bag X05, Onderstepoort 0110, South Africa
[4]South Africa Department of Microbiology and Plant Pathology, University of Pretoria, Pretoria, South Africa
[5]Onderstepoort Veterinary Institute-Transboundary Animal Diseases Programme (OVI-TADP), Onderstepoort, Gauteng, South Africa
[6]Current address: Bill and Melinda Gates Foundation, Seattle, USA

S1 Sequencing methods

More details on the experimental setup can be found in [1] and [2]. The samples included micro-dissected tissues (pharyngeal tonsil, palatine tonsils and dorsal soft palate) from three buffaloes obtained at 35 or 400 days post-infection with FMDV, which were sequenced by Sanger technology. RNA was extracted from LMD material using RNeasy Micro kit (Qiagen), followed by cDNA synthesis using TaqMan RT reagents (Agilent) and random hexamers, then the VP1 region of SAT1 was amplified using Platinum Taq Hi-Fidelity (Invitrogen) and the following primer pair: 5'-AGTGCTGGACCCGACTTCGA-3' and 5'-TGTAGCGATCCTTGCCACCGT-3' and the VP1 fragment was cloned into a TOPO®TA vector (Life Technologies). After colony picking and plasmid purification, the fragments were Sanger sequenced using BigDye terminator v3.1 (Applied Biosystems) and M13 primers.

The inoculum used for the experiment, as well as six further samples obtained from tonsil swabs and probangs of four animals culled between 200 and 400 days post inoculation, were sequenced at high throughput. RNA was extracted using RNeasy mini Kit (Qiagen), followed by cDNA synthesis using SuperScript™ III First-Strand Synthesis System (Life Technologies), amplification of the capsid region using Platinum Taq Hi-Fidelity (Invitrogen) and the primer pair 1A1F/2B2R (sequences available on request). Libraries were constructed using Nextera XT DNA Sample Preparation Kit

*Email: luca.ferretti@gmail.com, luca.ferretti@bdi.ox.ac.uk

(Illumina) and deep sequenced on a MiSeq system using 300 cycle version 2 reagent cartridges (Illumina) to produce paired end reads of approximately 150 bp each.

S2 Genetic content of the inoculum

After the removal of reads from contaminations, all sequenced FMDV reads belong to the SAT1 serotype. The consensus sequence of VP1 is very similar to the sequence of SAT1/KNP/196/91. There are 26 SNPs at frequency $> 1\%$ in VP1, of which 22 have an allele at frequency about 0.45. This corresponds to a strong haplotype structure in the inoculum, with at least two main variants (or better, two swarms around these variants, plus their recombinants) differing by about 3% in their VP1 sequence. The majority of these mutations are at the third codon position (17 out of 26, $p = 4 \times 10^{-3}$ by multinomial test), suggesting purifying selection pressure on the virus.

S3 Error model for Sanger sequences

All sequences obtained by Sanger sequencing from two animals culled at 35 dpi and one culled at 400 dpi were pooled together and aligned to the sequence of the inoculum using MAFFT-ginsi [3]. From this alignment, the distribution of the counts of all minor alleles in the sample was extracted. Since the distribution of sequencing errors was not known, we fitted an error model to the low-frequency part of the allele distribution, under the conservative assumption that all low-frequency alleles were sequencing errors. Simple models of random errors predict a Poisson distribution of error frequencies (resulting from rare, independent errors in each sequence) and more generally the tail of the error distribution is expected to be exponential. In fact the distribution of alleles present in three to ten sequences was well-fitted by a geometric distribution $P(c) = 186 \cdot 0.69^{(c-1)}$ (Figure A), which is the discrete version of an exponential distribution. From this geometric fit, the number of expected false SNPs in the sample above a threshold count $\bar{c}$ was estimated as $E[\text{false SNPs}] = 186 \cdot 0.69^{\bar{c}}/(1 - 0.69)$. We fix the threshold by requiring that $\mathrm{E}[\# \text{ false SNPs}] < 0.5$, that implies $\bar{c} = 20$.

S4 Linkage disequilibrium D' from sequences and short reads

Consider two biallelic variables sites in the FMDV genome, with alleles A, a at the first site and B, b at the second one. The Linkage Disequilibrium D for multiple haploid sequences is defined in terms of allele frequencies at both sites by the standard expressions [4]

$$D = f_{Aa} - f_A f_a = f_{Aa} f_{Bb} - f_{Ab} f_{aB} \tag{S1}$$

The linkage disequilibrium D is not normalised, i.e. it covers a wide range of values even for perfectly linked mutations without recombination. To take this into account, we consider the normalised value D'

$$D' = \begin{cases} \frac{D}{\max_{f_{Aa}}(D)} & D \geq 0 \\ \frac{D}{|\min_{f_{Aa}}(D)|} & D < 0 \end{cases} \tag{S2}$$

which is always ± 1 in the absence of recombination, provided that the variants are not generated by recurrent mutations.

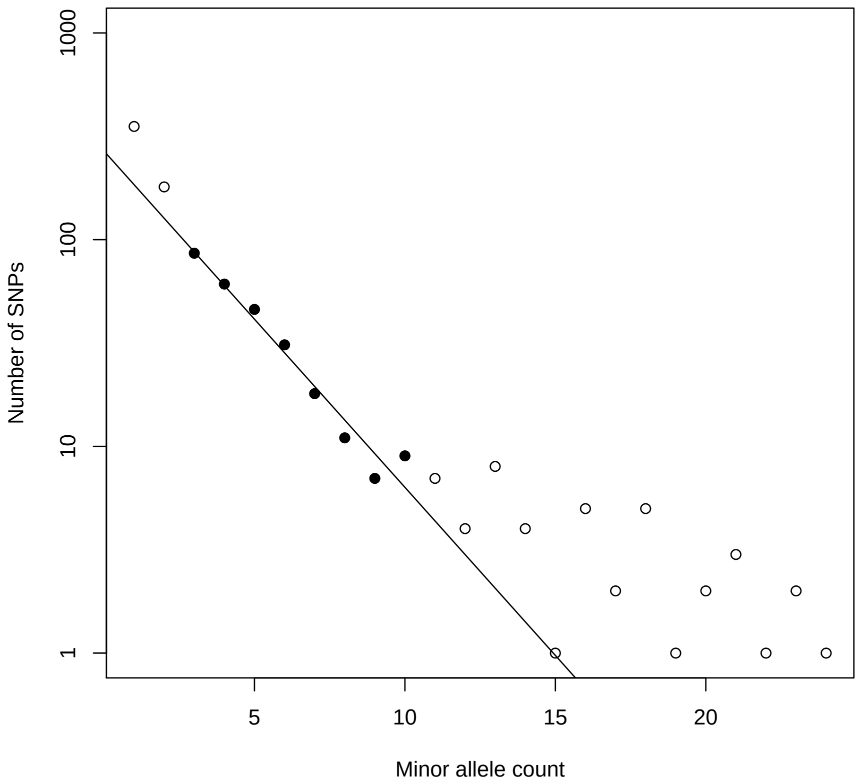

Figure A: The low-frequency part of the distribution of minor allele counts. The data with counts between 3 and 10 (black dots) were fitted by an exponential model (black line)

For a finite sample, If there are c reads or sequences covering both SNVs, it is possible to estimate the linkage disequilibrium between their alleles by restricting the analysis on these c sequences and estimating D' based on the sample frequencies. The result of this computation for inter-swarm SNVs in the inoculum is shown in Figure B.

Since the sample frequencies have an uncertainty of order $\sqrt{\frac{f(1-f)}{c}}$ with respect to the actual frequencies, the computation of LD from a finite sample results in an error of order $1/\sqrt{c}$. More precisely, for pairs of linked loci of similar frequency q and covered by c reads, the sampling error on the estimates of D' [5] is about

$$\sigma^2(D') = \frac{(1-D')(1+(\frac{1}{q(1-q)}-3)D'+2D'^2)}{c} \tag{S3}$$

This error is illustrated in Figure CA,B for the deep sequencing of the inoculum as well as for all viral Sanger sequences from buffaloes.

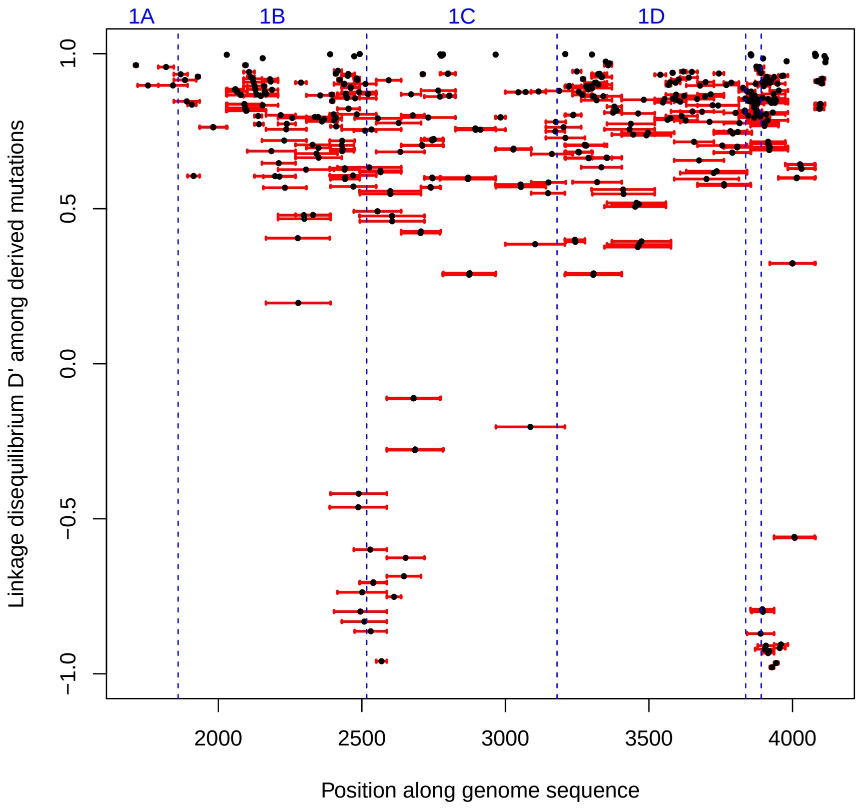

Figure B: Normalised linkage disequilibrium D' between pairs of derived swarm-specific variants covered by at least 10^4 reads. Red bars illustrate the interval between variants, with a black dot at the mid-point.

S5 Inference of recombination and related uncertainties

The advantage of LD is that it takes naturally into account the fact that many recombination events are invisible, i.e. they do not alter D since they occur between two sequences with the same combi-

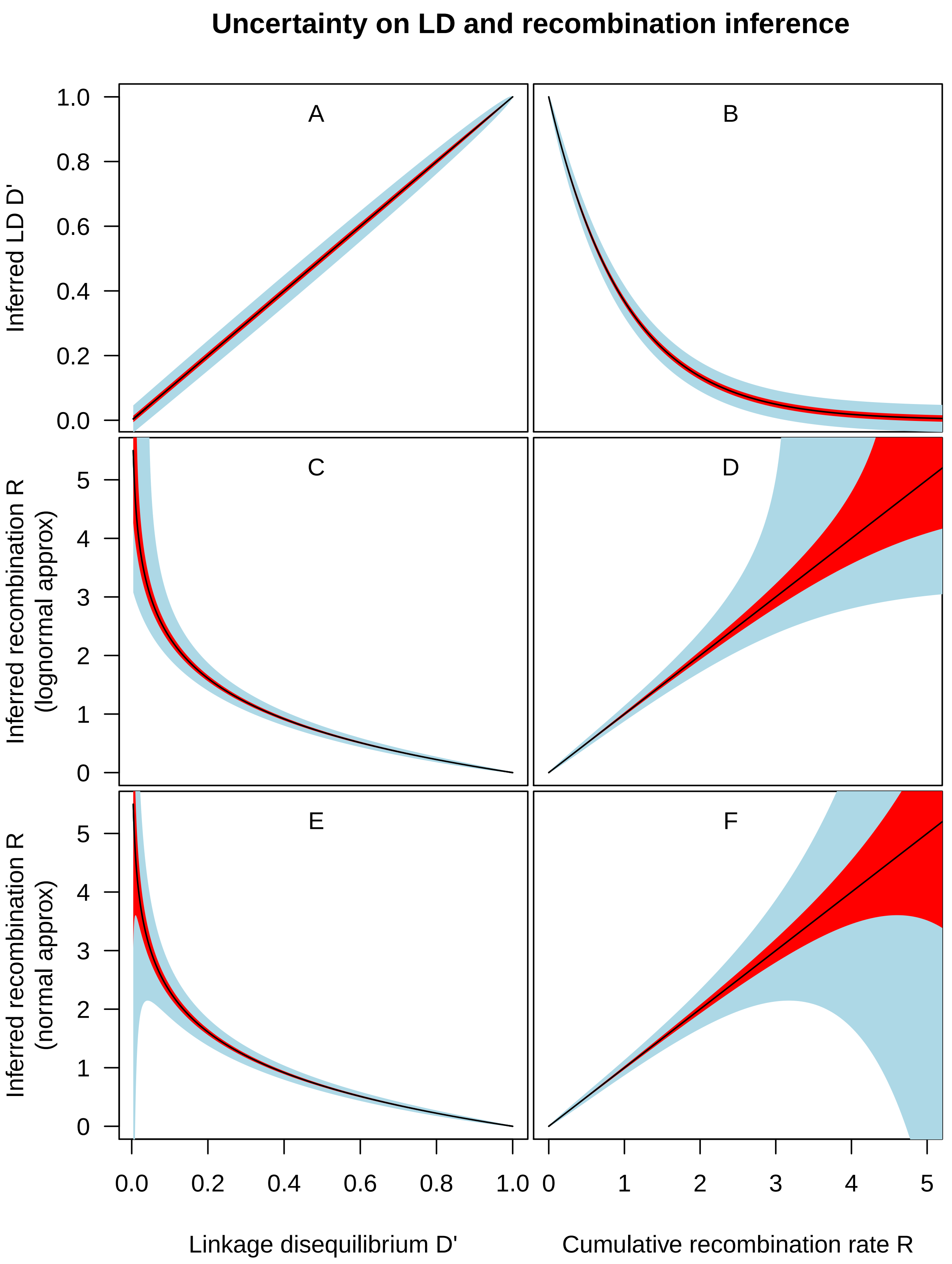

Figure C: Uncertainty at 1σ level on inferred LD and recombination rates. Approximated via the LD uncertainty (C,D) or with equation S7 (E,F). Red: deep sequencing data; grey: Sanger sequences.

nation of alleles. In fact, its decay in time follows the law

$$\mathrm{E}\left[\frac{dD}{dt}\right] = -rD \tag{S4}$$

where r is the recombination rate per sequence between the two loci. Hence, for variants initially in complete linkage,

$$\mathrm{E}\left[D'\right] = e^{-rt} = e^{-R} \tag{S5}$$

where R is the cumulative recombination rate in time. For this reason, we can estimate the recombination rate by inverting the previous equation:

$$\hat{R} = -\log(D') \tag{S6}$$

The uncertainty on $\hat{R}$ can be estimated either by assuming an approximately Gaussian distribution for D', as illustrated in Figure CC,D, and transforming the Gaussian confidence intervals for D' into the corresponding confidence intervals for R. Alternatively, the delta method implies that the variance of the estimate of $R = -\log(D')$ is about

$$\sigma^2(R) \approx \frac{\sigma^2(D')}{D'^2} = \frac{(1-D')(1+(\frac{1}{q(1-q)}-3)D'+3D'^2)}{cD'^2} \tag{S7}$$

illustrated in Figure CE,F. For values of $R > 0.05$, the relative error $\sigma(R)/R \lesssim 10\%$ is reasonably small. Even for smaller values of $R > 0.01$, the error $\sigma(R)/R \lesssim 25\%$ is under control.

Note that for large recombination rates, there is a saturation effect with smaller and smaller changes in LD as the recombination increases. This corresponds to the exponential dependence in equation S5. The consequence is a strong increase in the uncertainties on recombination rates for values of cumulative recombination greater than 2, or D' values lower than 0.2.

In this experiment, typical values of D' among reasonably close SNVs are large enough that this saturation effect does not affect our data, as can be seen from Figure B.

Errors on recombination rates are shown explicitly in Figure D by mapping the 1σ confidence intervals of D'. For VP1 sequences in buffalo, the evidence of recombination in terms of reduction in LD compared to complete linkage is also significant for all pairs of SNVs, as illustrated by the z-scores in Figure E.

The above approach is denoted by "local" approach in the text and it is based on the above estimate (S6) for consecutive variants only. The second approach that we propose is denoted as "global" approach and is given by the weighted least squares [6] estimate R^{wls} from all variants. More precisely, it is defined by the equations:

$$\sum_j \sum_{I \supset i,j} R_j^{\text{wls}}/\mathrm{Var}(\hat{R}_I) = \sum_{I \supset i} \hat{R}_I/\mathrm{Var}(\hat{R}_I) \tag{S8}$$

where i, j denote intervals between consecutive variants and I intervals between any pair of variants. For this estimator, we use the approximate form for the variance: $\mathrm{Var}(\hat{R}) = (D')^{-2}/c + \hat{R}$, where c is the number of reads covering both variants in the pair; the first term comes from the delta method applied to the variance of binomial sampling of c sequences (assuming low recombination and similar frequencies for all SNVs), the second from the Poisson noise of the random recombination events. To get comparable results between Sanger and short-reads data, only intervals of length less than 200 bases are used for the "global' estimate for analyses involving both approaches.

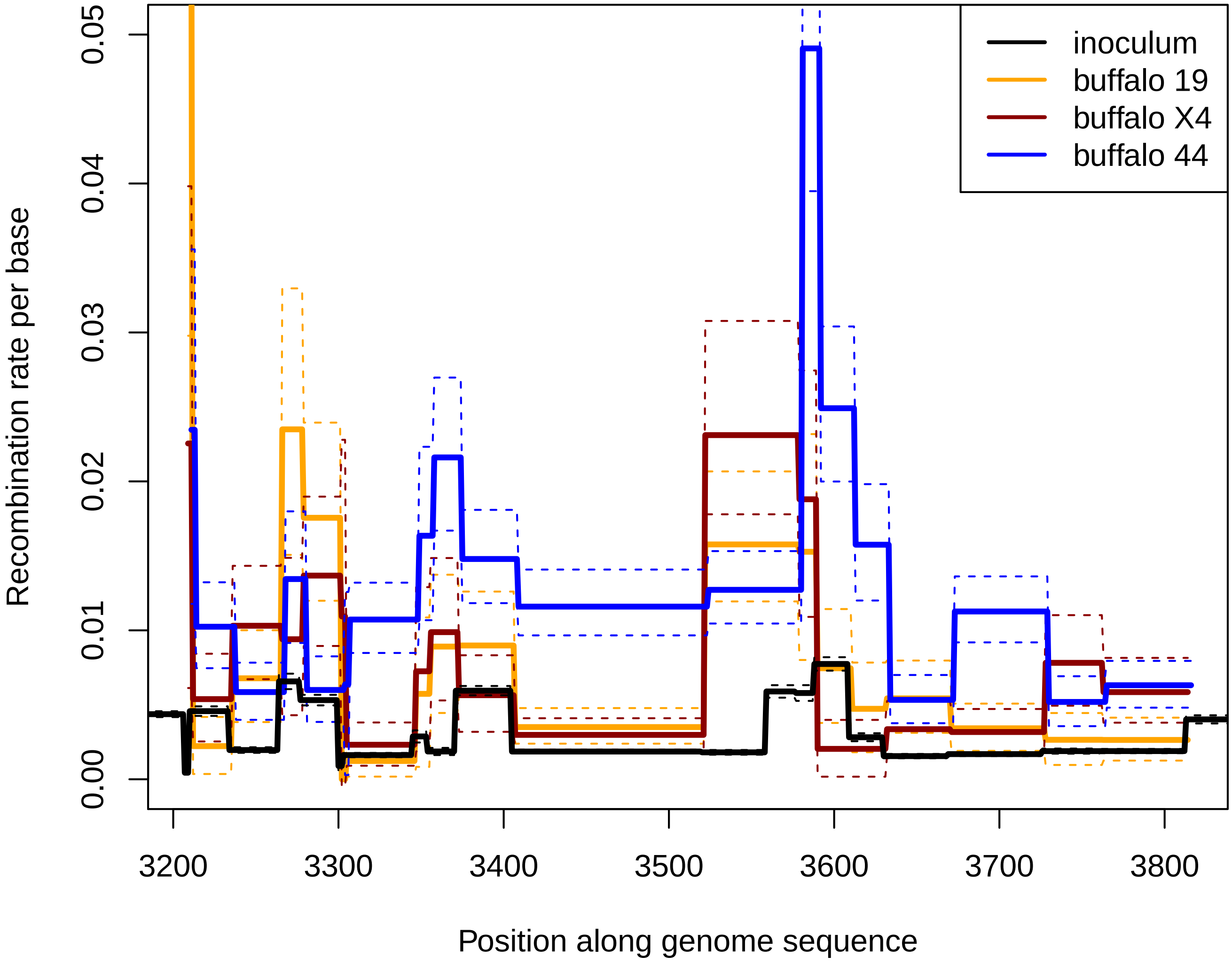

Figure D: Inferred cumulative recombination rates per base. Dashed lines illustrate the 1σ uncertainties.

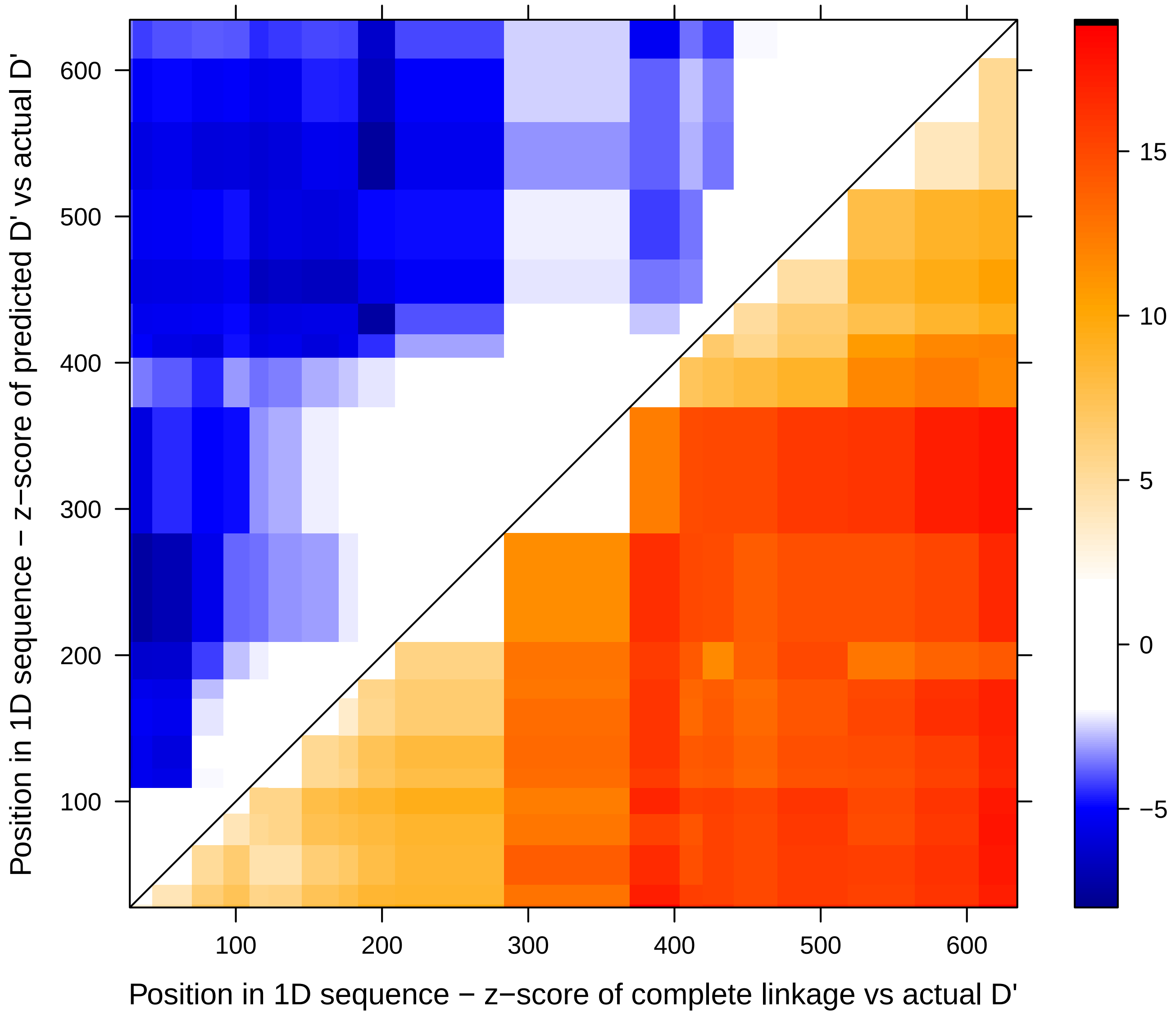

Figure E: Relative z-scores with respect to D', i.e. $z(x) = \frac{x-D'}{\sigma(D')}$, computed across all sequences from buffalo tissues. Note that z-scores between -2 and 2 are in white. Lower triangle: complete linkage $(D' = 1)$ z-scores are all larger than 2, proving that LD is significantly lower than complete LD for all pairs of mutations. Upper triangle: most predicted D' z-scores are less than -2, showing that epistasis affects significantly LD among most mutations.

S6 Multi-swarm structure

Deep sequencing of inoculum

The description of the peculiar structure of the quasi-species is based on three pieces of evidence:

- The distribution of minor allele frequencies in the inoculum is strongly bimodal (Figure 2A in the Main Text), with a expected tail of low-frequency alleles disappearing before frequency 0.2, and a large number of intermediate-frequency alleles peaked at 0.4. This already is a strong hint of haplotype structure. The derived alleles show a similar distribution of frequencies and the intermediate-frequency SNPs are distributed uniformly along the sequence (Figure 2B in the Main Text).
- A haplotype structure is defined by a strong linkage disequilibrium (LD) among derived alleles. In the inoculum, LD can be measured only between close SNPs. Figure 2B in the Main Text shows that the LD between the derived alleles of consecutive SNPs is very strong ($D' \approx 1$), therefore supporting a local haplotype structure. The few mutations with $D' \approx -1$ point to an erroneous inference of their ancestral state.
- The Sanger sequences from microdissections of infected buffalos were sampled after the acute phase of the infection, hence they are affected by selection and recombination in a complex way. However, when looking at the SNPs already present in the inoculum, they still show a bimodal distribution of frequencies (Figure F) and signatures of a strong haplotype structure (Figure 3 in the Main Text).

These data support a strong haplotype structure of the quasi-species, with clouds of genotype concentrated around two well-defined haplotypes and their recombinants. The frequency of these haplotypes in VP1 can be estimated by taking the average of the frequencies of the derived and ancestral variants in the SNPs, obtaining 0.44 and 0.56 respectively.

The minor haplotype among the buffalo sequences in Figure 3 does not correspond to any of the two main haplotypes of the quasi-species. It is very similar to the major haplotype of the inoculum, but with two "fixed" variants identical to the minor haplotype. Nevertheless, since it is observed at similar frequencies in the different individuals and tissues, it should have been present in the inoculum: otherwise, if it would have emerged as a random recombinant, its evolutionary outcomes would have been highly stochastic. An estimate of the initial frequency of this haplotype in the inoculum can be obtained by the fraction of reads that (i) cover one of the two sites with "fixed" variants and a neighbouring SNP and (ii) are compatible with this haplotype. The estimated fraction of reads satisfying both conditions (i),(ii) among all reads satisfying condition (i) is about 0.02.

Viral sequences from micro-dissections

The frequencies of all SNPs among the viral sequences from micro-dissection are shown in Figure F. Their distribution is again bimodal. New SNPs (corresponding to monomorphic sites in the VP1 sequence of the inoculum) do not show any significant purifying selection ($p > 0.05$ by multinomial test on their codon position).

Two main haplotypes (plus several recombinants) are present among the sequences post-inoculation. The major haplotype is the same as the minor haplotype in the inoculum. Instead, the second main haplotype post-inoculation is not the major haplotype of the inoculum, but it is rather a (possibly recombinant) variant of that haplotype differing only by 0.2% in the VP1 sequence. The VP1 sequences of the minor haplotype in the inoculum and of this recombinant differ in 20 positions out of

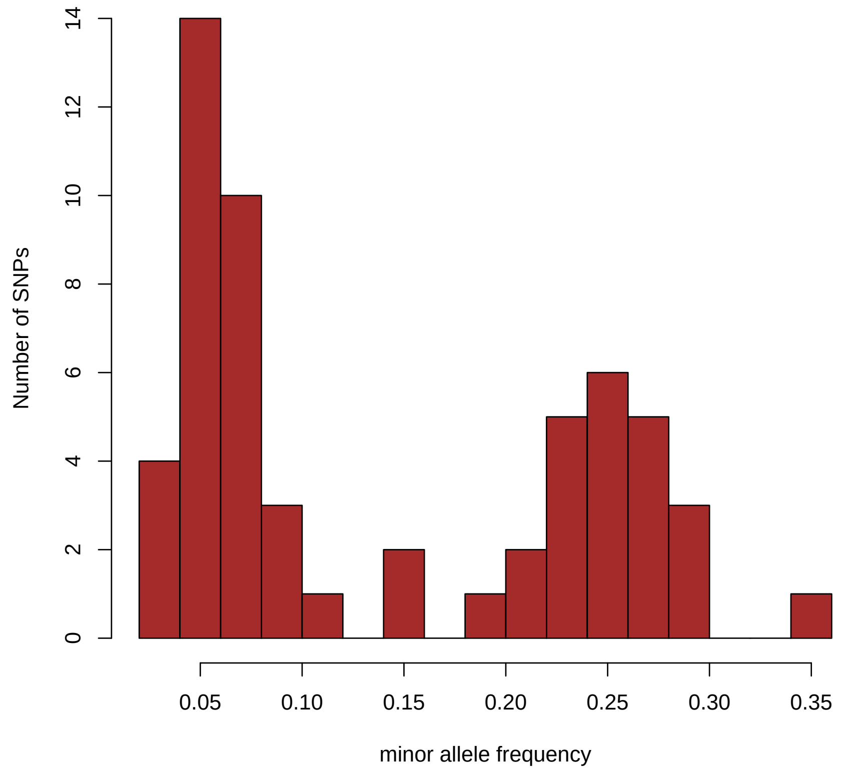

Figure F: Distribution of SNP frequencies in viral sequences from all micro-dissections of buffalo tissues.

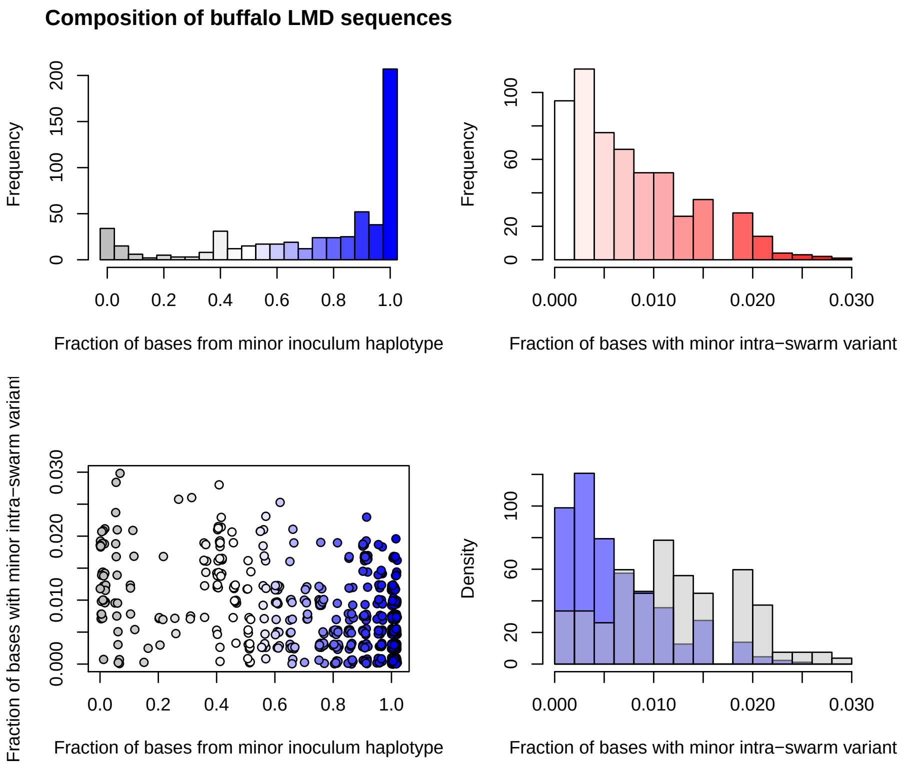

Figure G: Left: distribution of the composition of each sequence in terms of the two swarms described in the paper. More precisely, it is the distribution of the fraction of SNVs that can be attributed to the minor swarm in the inoculum (top), computed across all viral sequences from microdissections of buffalo tissues. The relation between sequence composition and the amount of minor intra-swarm variants is also illustrated (bottom). Right: Distribution of abundance of minor intra-swarm SNVs within each sequence, for both swarms (top) and separated by swarm (bottom).

the 22 SNPs at intermediate frequency in the inoculum, while the remaining two SNPs at intermediate frequency in the inoculum are fixed post-inoculation. Assuming that this variant was already present in the inoculum - which is the most parsimonious hypothesis - it is possible to estimate its initial frequency at about 2% or less in the inoculum, as discussed above. Note that the value of linkage disequilibrium D among the remaining variants is affected by these changes in frequency post inoculation, but its normalized value D' is not.

A more detailed picture of the two quasi-species and their recombinants in buffalo, as well as the diversity within swarms, is illustrated in Figure G. From these figures, it is apparent that roughly half of the sequences are derived from a single swarm; however, the other half is constituted by recombinants, containing contributions from both swarms in different proportions. Intra-swarm variants contribute to overall sequence diversity roughly as much as the inter-swarms SNVs discussed before. Sequences belonging to the major swarm within buffalo tissues contain less intra-swarm variants, which could be explained by a rapid growth in the relative size of this swarm even before inoculation.

S7 Biases in recombination inference

Our approach to the inference of recombination rates between nearest neighbour pairs of variants can be biased by several biological factors:

- Local genetic structure of the swarm: even if the population is composed of a mixture of the two swarms with frequencies q and $1-q$, there could be local inhomogeneities that cause one or the other swarm to be locally more abundant. In such cases, the likelihood of two viruses from two different swarms infecting the same cell could be reduced with respect to the well-mixed case $\pi = 2q(1-q)$. If the average local genetic diversity is $\pi_{local} = \mathrm{E}[2q_{local}(1-q_{local})]$, the local genetic structure has an impact quantified by $1 - F_{st}^{local} = \pi_{local}/\pi$.
- Viability of recombinants: recombination can generate defective viral sequences, e.g. due to deletions caused by the recombination process. Such viruses are unlikely to replicate. We assume that these non-viable sequences are generated with probability p_{nv} and are removed at a rate σ_{nv}.
- Epistatic interactions between nearest neighbour pairs of variants: such interactions cannot be detected, since they occur at the resolution of the finest scale available in the experiment. Recombinants are likely to be less fit that the original swarms: we denote the fitness cost of recombinants by s.

To understand the quantitative impact of these effects, we describe mathematically the evolution of the amount of visible recombinants. The equations for the evolution of the fraction of viable and non-viable recombinants are

$$\frac{df_{r,v}}{dt} = r_{i,j}\pi_{local}(1-p_{nv}) - s_{i.j}f_{r,v} \tag{S9}$$

$$\frac{df_{r,nv}}{dt} = r_{i,j}\pi_{local}p_{nv} - \sigma_{nv}f_{r,nv} \tag{S10}$$

If the observed recombination is weak, then it can be well approximated by

$$r_{i,j}^{observed} \approx \frac{1-D'_{i,j}}{\Delta t} = \frac{f_{r,v}+f_{r,nv}}{2q(1-q)\Delta t} = r_{i,j}(1-F_{st}^{local})\left[p_{nv}\frac{1-e^{-\sigma_{nv}\Delta t}}{\sigma_{nv}\Delta t} + (1-p_{nv})\frac{1-e^{-s_{i,j}\Delta t}}{s_{i,j}\Delta t}\right] \tag{S11}$$

From this expression it is clear that the local genetic structure suppresses the observed recombination rate by a factor $1-F_{st}^{local}$. From the results in [2], we estimate that $1-F_{st}^{local} \gtrsim 0.8$, hence local genetic structure could be responsible for a modest suppression of recombination of up to 20%. The impact of epistatic interactions is relevant only when defective recombinants are not exceedingly probable, otherwise the suppression of recombination depends on the lifetime $1/\sigma_{nv}$ of non-viable recombinants.

If the recombinants are not dominantly defective, i.e. if $p_{nv} \lnapprox 1$, we can assume $\sigma_{nv} \gg s$ and therefore neglect the contribution of non-viable recombinants, approximating the result as

$$r_{i,j}^{observed} \approx r_{i,j}(1-F_{st}^{local})(1-p_{nv})\frac{1-e^{-s_{i,j}\Delta t}}{s_{i,j}\Delta t} \tag{S12}$$

In this case, all three factors contribute to the suppression of the observed recombination rates.

It should be pointed out that for many applications, the effective recombination rate that matters is the one that takes into account the local genetic structure and the non-viable recombinants as well,

since these effects occur on a very short scale in space and time and are therefore unavoidable. Hence, the effective recombination rate that matters is

$$r_{i,j}^{effective} = r_{i,j}(1 - F_{st}^{local})(1 - p_{nv}) \approx r_{i,j}^{observed} \frac{s_{i,j}\Delta t}{1 - e^{-s_{i,j}\Delta t}} \quad \text{(S13)}$$

which is larger than the inferred rates because of the epistatic effects. As we will show later, recombination can be typically enhanced by $25\% - 100\%$ with respect to the observed one, since epistatic interaction coefficients tend to lie in the range $s\Delta t \sim 0.5 - 1.5$.

S8 Epistasis from Linkage Disequilibrium

Detecting epistasis

In the absence of epistasis, the Linkage Disequilibrium is expected to decay exponentially with recombination, i.e. $D' = e^{-R}$. Recombination can be modelled as an inhomogeneous Poisson process, hence by the superposition properties of Poisson processes, the recombination rate between two variants is the sum of all recombination rates between consecutive bases in the genomic interval defined by the two variants, i.e. for variants in position x and y, the equation

$$R_{x,y} = \sum_{z=x}^{y-1} R_{z,z+1} \quad \text{(S14)}$$

should be satisfied. We can use these equations to detect the presence of epistasis among our variants.

From the ith and jth variant ($i < j$) and the inferred recombination rate between them $R_{i,j} = -\log(D'_{i,j})$, we define the predicted recombination rates as

$$R_{predicted} = \sum_{k=i}^{j-1} R_{k,k+1} \quad \text{(S15)}$$

The suppression of recombination $R - R_{predicted} = log(D'_{predicted}/D')$ between all pairs of SNVs represents the impact of epistasis among these variants and it is shown in Figure 7 in the Main Text. The relative suppression $\frac{R-R_{predicted}}{R_{predicted}}$ reaches up to -74%, as shown in Figure H (lower triangle).

The detection of epistatic effects among VP1 sequences from buffalo is robust with respect to statistical and sampling uncertainties. In fact, z-scores of the difference between observed and predicted LD in Figure E (upper triangle) are much lower than -2 for most pairs of variants.

It is worth noticing that this approach would not work for viral populations with a strong subpopulation structure among different tissues or niches. If that would be the case, LD could be caused by different linked variants within each subpopulation and would not decay exponentially. In this experiment, this caveat does not apply since population structure is weak, as shown in details through genetic differentiation analyses in [2].

Heuristic inference of pairwise epistatic interactions

Reduction in recombination rates between two mutations can be due to direct epistatic interactions between those mutations, or indirect effect from epistatic interactions between other linked variants. Here we present a heuristic approach to extract the most relevant pairwise epistatic interactions.

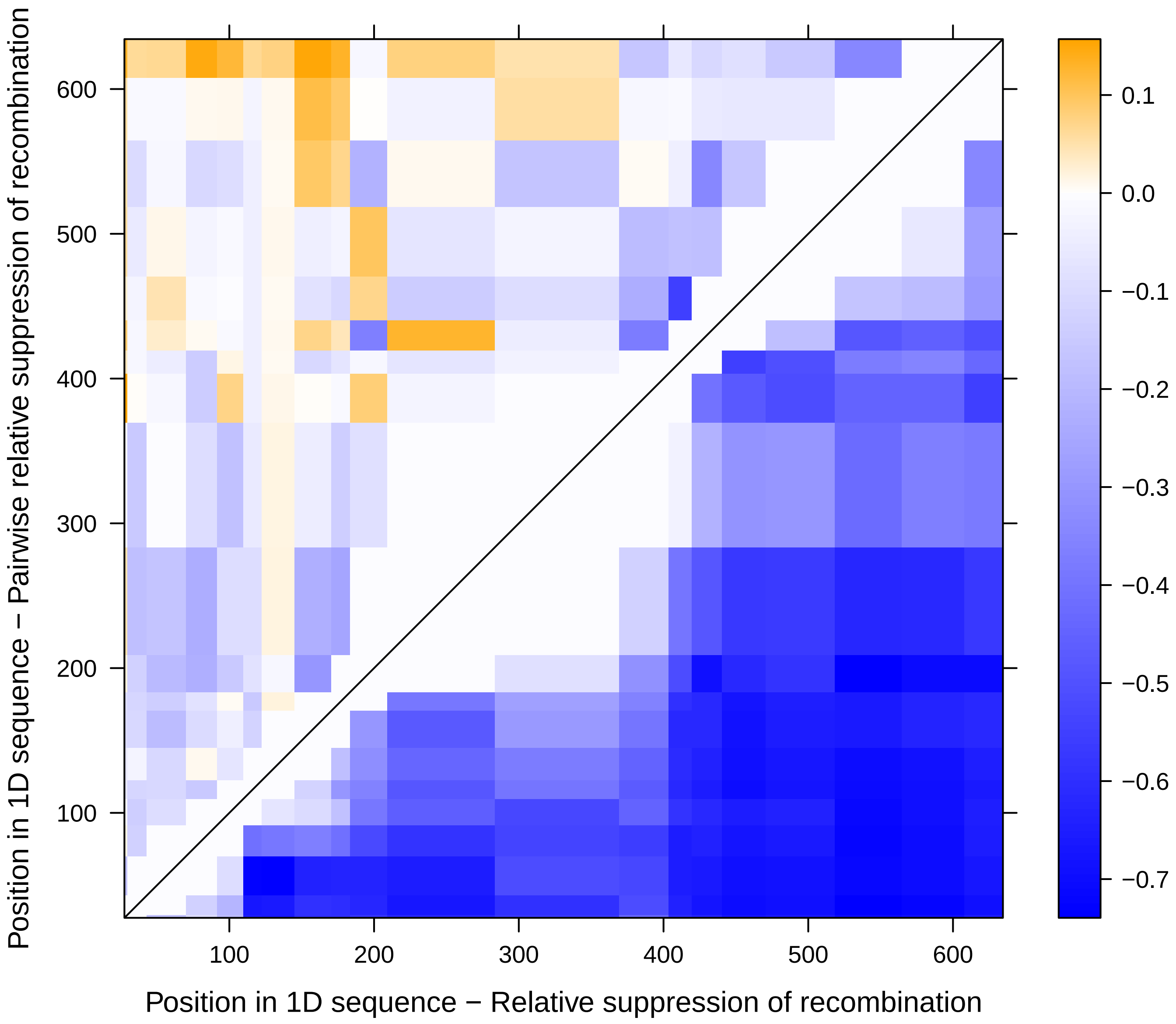

Figure H: Suppression of recombination for all sequences from buffalo tissues. Lower triangle: relative suppression of recombination $(R - R_{predicted})/R_{predicted}$ with respect to the predictions from local recombination rates. Upper triangle: relative suppression of recombination $(R - R_{2,predicted})/R_{2,predicted}$ with respect to the heuristic predictions corrected to extract only the effect of pairwise direct interactions.

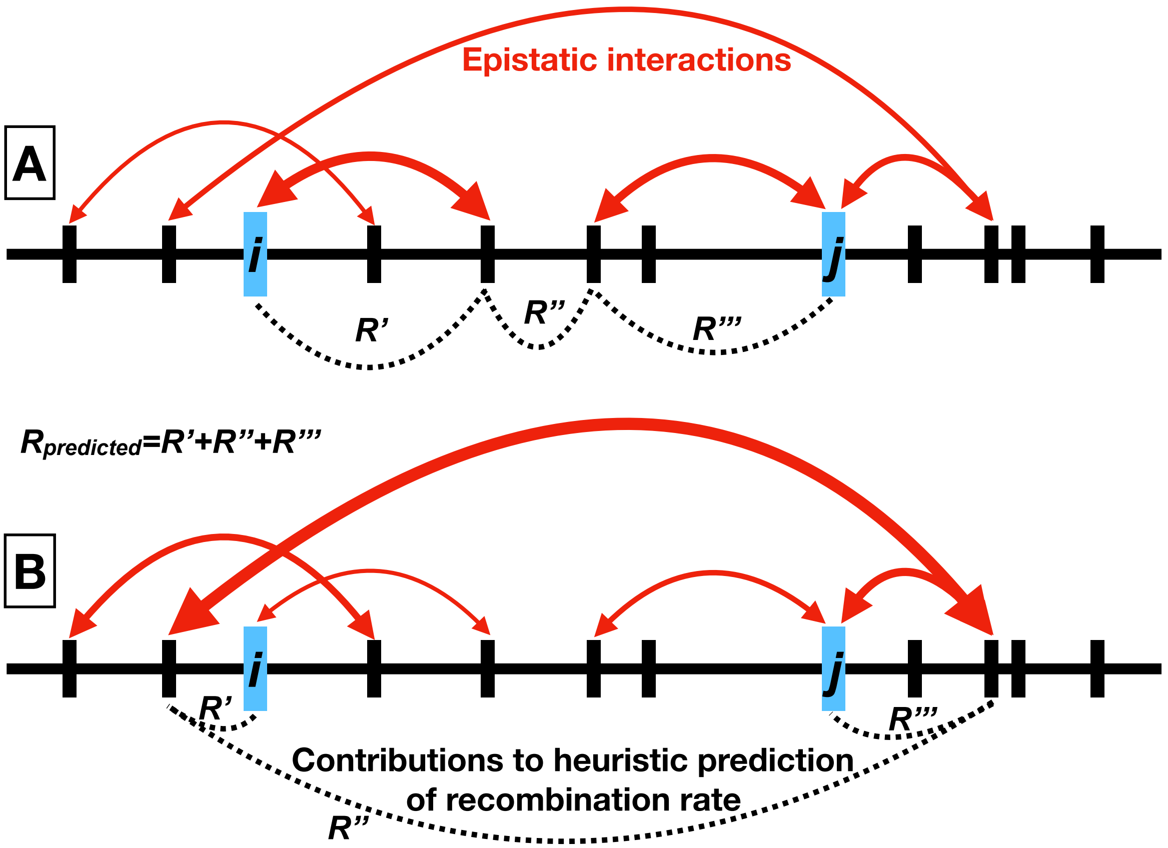

Figure I: Illustration of multiple epistatic interactions among variants along a sequence (red arrows) and of the corresponding heuristic prediction for $R_{2,predicted}$ between variants i and j (dashed lines). In both examples A and B, the prediction is $R_{2,predicted} = R' + R'' + R'''$, which is the chain of recombination rates between i and j with the minimum sum. As shown by this illustration, the heuristic prediction $R_{2,predicted}$ accounts for most indirect effects of other epistatic interactions on the LD between i and j.

The idea is to compare the recombination rate inferred from LD between two variants with the cumulative recombination rates across the most linked chain of variants connecting the two. The idea is illustrated in Figure I.

More precisely, from the ith and jth variant and the inferred recombination rate between variants $R_{k,k'} = -\log(D'_{k,k'})$, we define the predicted recombination rates as

$$R_{2,predicted} = \min_{\{k_1,\dots k_n\}} \left(R_{i,k_1} + \sum_{a=1}^{n-1} R_{k_a,k_{a+1}} + R_{k_n,j} \right) \tag{S16}$$

where the ordered set $\{k_1, \dots k_n\}$ contains at least one variant and all variants in it are different from i and j. This heuristic prediction is able to capture the effect of one or a few epistatic interactions between variants linked to i and j, provided that there is a dominant chain among such interactions (see Figure I).

The relative pairwise suppression of recombination $(R - R_{2,predicted})/R_{2,predicted}$ is shown in Figure H (upper triangle). Direct epistatic interactions appear to suppress recombination up to -55%.

Note that when there is no direct epistatic interaction among two variants but there are strong epistatic interactions between linked variants, $R_{2,predicted}$ could even be smaller than the observed R. This does not necessarily mean that there are "negative" epistatic interactions, and in fact there is no evidence for such significant "negative" interactions in Figure E (upper triangle).

Inference of selection strength for epistatic interactions

The suppression of recombination is related to the selection coefficients, but it is not a direct measure of their strength. To infer the actual strength of the epistatic selection coefficients, we need an explicit model relating them to the suppression of recombination.

Consider two sites with variants of fixed frequency q, recombining with a recombination rate r. Assume also that all recombinants have a fitness disadvantage $-s$. Initially, the two sites are fully linked and $D' = 1$. The expected evolution of recombinants between these sites can be written in terms of a single differential equation for the fraction of recombinants f_r:

$$\frac{df_r}{dt} = r(2q(1-q) - f_r) - s f_r (1 - f_r) \tag{S17}$$

which can be translated into an equation for the evolution of the linkage disequilibrium $D' = 1 - \frac{f_r}{2q(1-q)}$:

$$\frac{dD'}{dt} = -rD' + s(1-D')[1 - 2q(1-q)(1-D')] \tag{S18}$$

In turn, this can be solved in terms of the overall effective recombination rate $R = -\log(D')$ as

$$R = -\log\left[1 - \frac{1 + s/r - \gamma \coth\left[\frac{\gamma r t}{2} + \frac{1}{2}\log\left(\frac{1+s/r+\gamma}{1+s/r-\gamma}\right)\right]}{4q(1-q)s/r}\right] \tag{S19}$$

where $\gamma = \sqrt{(1+s/r)^2 - 8q(1-q)s/r}$.

In this formula, we can replace the recombination rate $r \cdot t \to R_{2,predicted}$ and the ratio $s/r = \frac{s \cdot t}{r \cdot t} \to s'/R_{2,predicted}$ and we end up with an implicit equation for the rescaled selection coefficient $s' = s \cdot t$:

$$\frac{4q(1-q)s'}{R_{2,predicted}}(1 - e^{-R}) = 1 + \frac{s'}{R_{2,predicted}} - \gamma \coth\left[\frac{\gamma R_{2,predicted}}{2} + \frac{1}{2}\log\left(\frac{1 + \frac{s'}{R_{2,predicted}} + \gamma}{1 + \frac{s'}{R_{2,predicted}} - \gamma}\right)\right] \tag{S20}$$

$$\gamma = \sqrt{(1 + s'/R_{2,predicted})^2 - 8q(1-q)s'/R_{2,predicted}} \tag{S21}$$

For numerical reasons, we estimate $s' = 0$ if the suppression of recombination is too small, i.e. if $R - R_{2,predicted} > -0.1$.

The results are shown in Figure J for all sequences and in Figures K,L,M for the different animals. The inferred coefficients s' corrected to estimate the strength of pairwise interactions, based on $R_{2,predicted}$, are shown in the lower triangle of Figure J. Most of the coefficients lie in the range $s' \sim 0 - 2$; if the replication time during the acute phase of the infection is a few hours, the selective coefficients can be estimated in the range $s \sim 0 - 0.1$.

S9 Direct Coupling Analysis from linkage disequilibrium among SNVs

The approach for the detection of direct epistatic interactions presented in the previous sections is grounded in population genetics for the inference of selection strengths, but is otherwise based on heuristic estimates of the predicted recombination rate corrected for indirect interactions. Here we present a different approach based on Direct Coupling Analysis [7]. Such approach is based on a simpler model for the probability to find a sequence as a function of its fitness and it is more appropriate for stationary ensembles of sequences, which are usually reached after longer evolutionary times. However, it has the great advantage of providing a simple and statistically grounded method to disentangle direct and indirect interactions.

Assume that we have SNVs denoted by indices $1 \dots s$ with alleles $a_1 \dots a_s$. Each allele can be represented by the values $a_i = -1$ if the variant belongs to the first swarm or $a_i = +1$ if it belongs to the second one. We assume that the population-scaled fitness of the sequence is given by the quadratic function

$$f(a_1 \dots a_s) = \sum_{i,j} J_{ij} a_i a_j + \sum_i h_i a_i \tag{S22}$$

where J_{ij} are direct epistatic couplings between variants. From standard population genetics arguments, the probability of a sequence is

$$P(a_1 \dots a_s) = \frac{e^{-f(a_1 \dots a_s)}}{Z} = \frac{1}{Z} \exp\left[-\sum_{i,j} J_{ij} a_i a_j - \sum_i h_i a_i \right] \tag{S23}$$

where $Z = \sum_{a_1} \dots \sum_{a_s} e^{-f(a_1 \dots a_s)}$ is a normalisation factor.

As a mathematical simplification, we can follow the Gaussian approximation approach proposed in [8], promoting the variables a_i to Gaussian random variables. Then Z can be computed explicitly as

$$Z = \frac{(2\pi)^{s/2}}{\sqrt{\det(J)}} \exp\left[\sum_{i,j} h_i J^{-1}_{ij} h_j / 4 \right] \tag{S24}$$

If the frequencies of the two swarms are q and $1-q$, the expected values are $\mathrm{E}[a_i] = 2q - 1$ and the variances are $\mathrm{Var}[a_i] = 4q(1-q)$. The covariances can be estimated as

$$\mathrm{Cov}[a_i, a_j] = \frac{\partial^2 \log Z}{\partial h_i \partial h_j} = \frac{J^{-1}_{ij}}{4} \tag{S25}$$

The LD is related to the covariances as follows. Assume positive linkage, i.e. $D > 0$. The definition of LD can be rewritten as

$$D_{ij} = \mathrm{Cov}\left[\frac{a_i + 1}{2}, \frac{a_j + 1}{2} \right] = \frac{J^{-1}_{ij}}{16} \tag{S26}$$

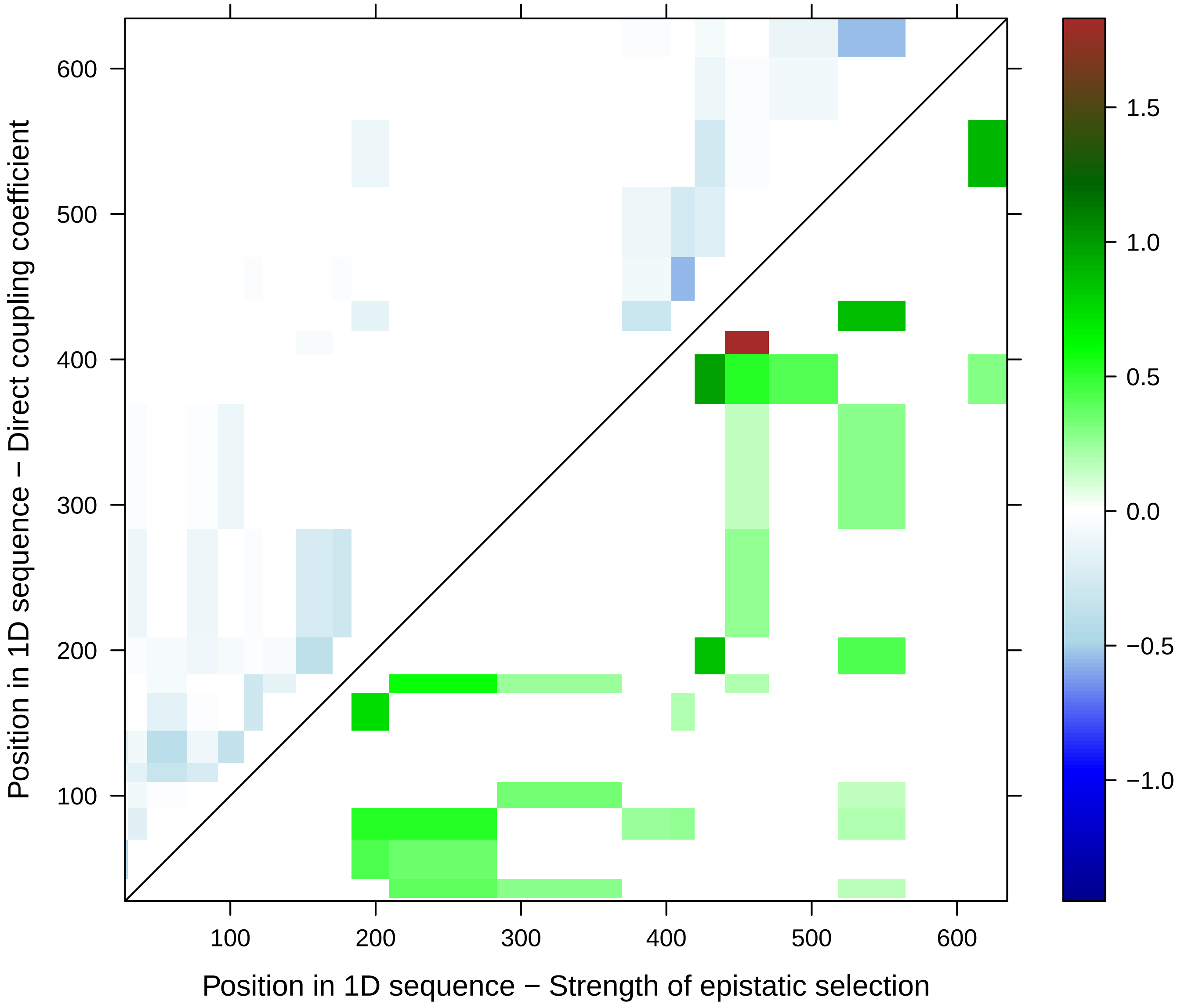

Figure J: Pairwise epistatic interactions inferred from all viral sequences from buffalo tissues. Lower triangle: Strength of selection coefficients s' for pairwise direct epistatic interactions inferred from the recombination R versus the predicted one $R_{2,predicted}$ corrected to extract only the effect of pairwise direct interactions. Upper triangle: pairwise direct coupling coefficients inferred from DCA.

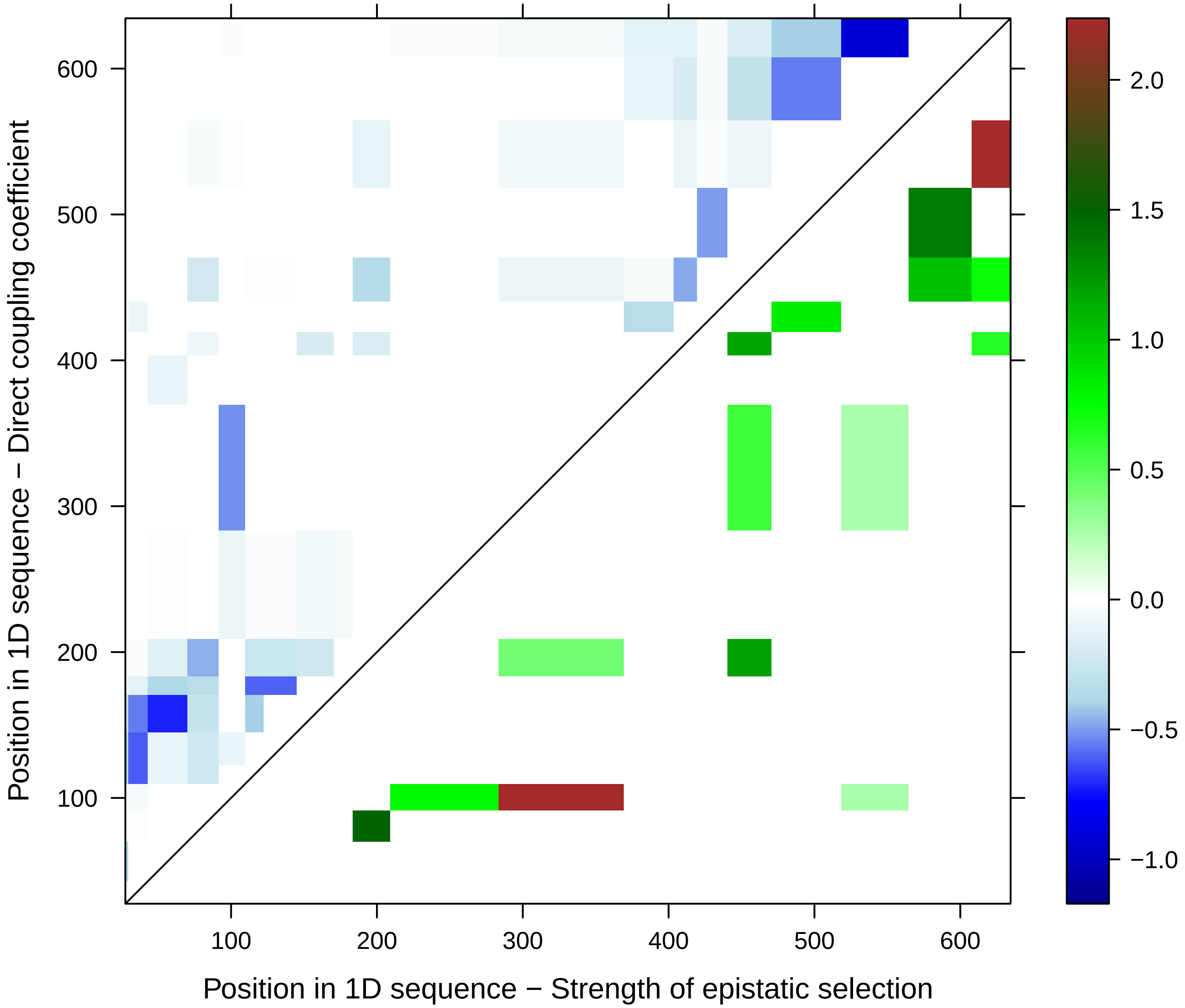

Figure K: Same as Figure J, but computed only from sequences from buffalo 19 (culled at 35 dpi).

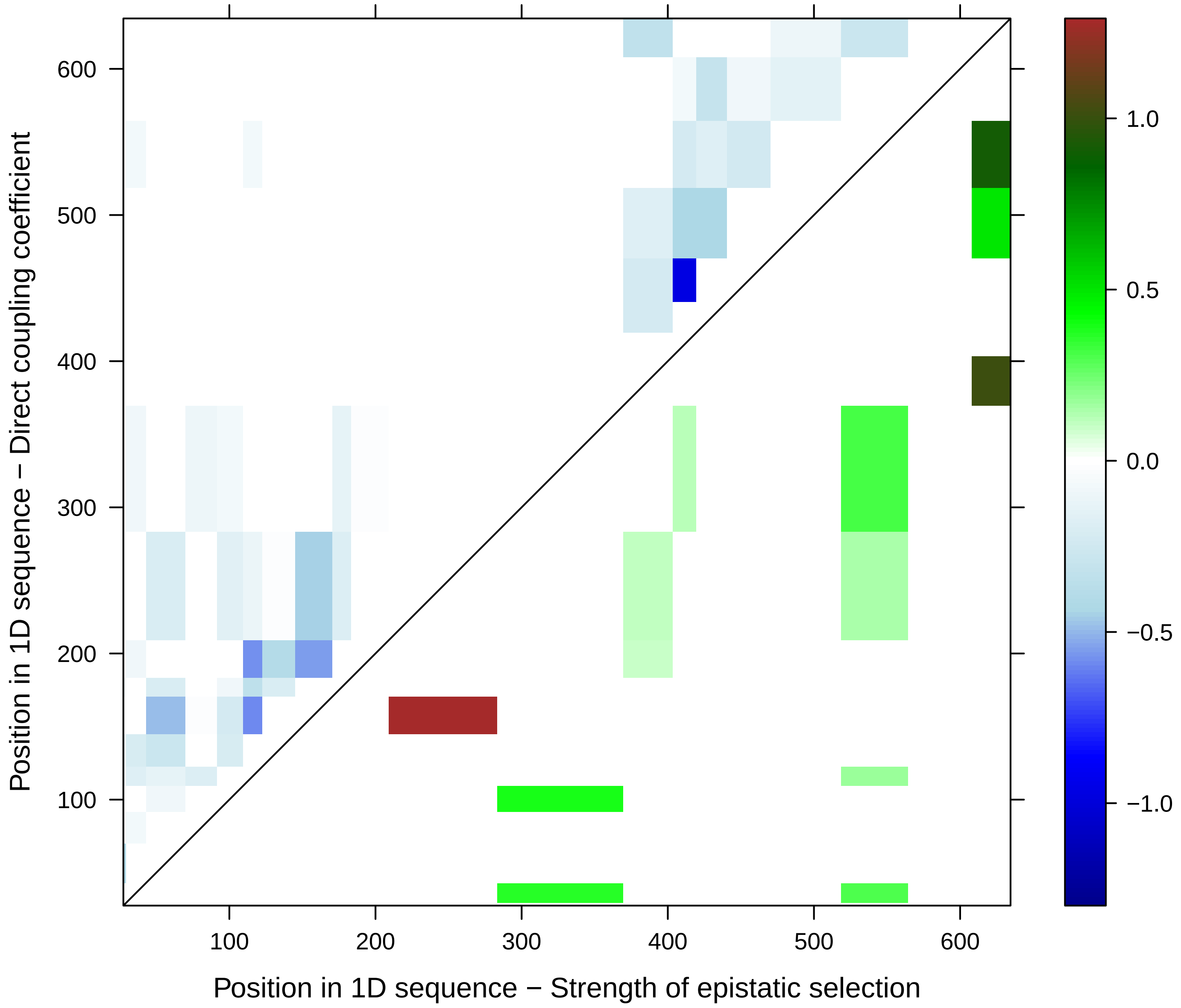

Figure L: Same as Figure J, but computed only from sequences from buffalo X4 (culled at 35 dpi).

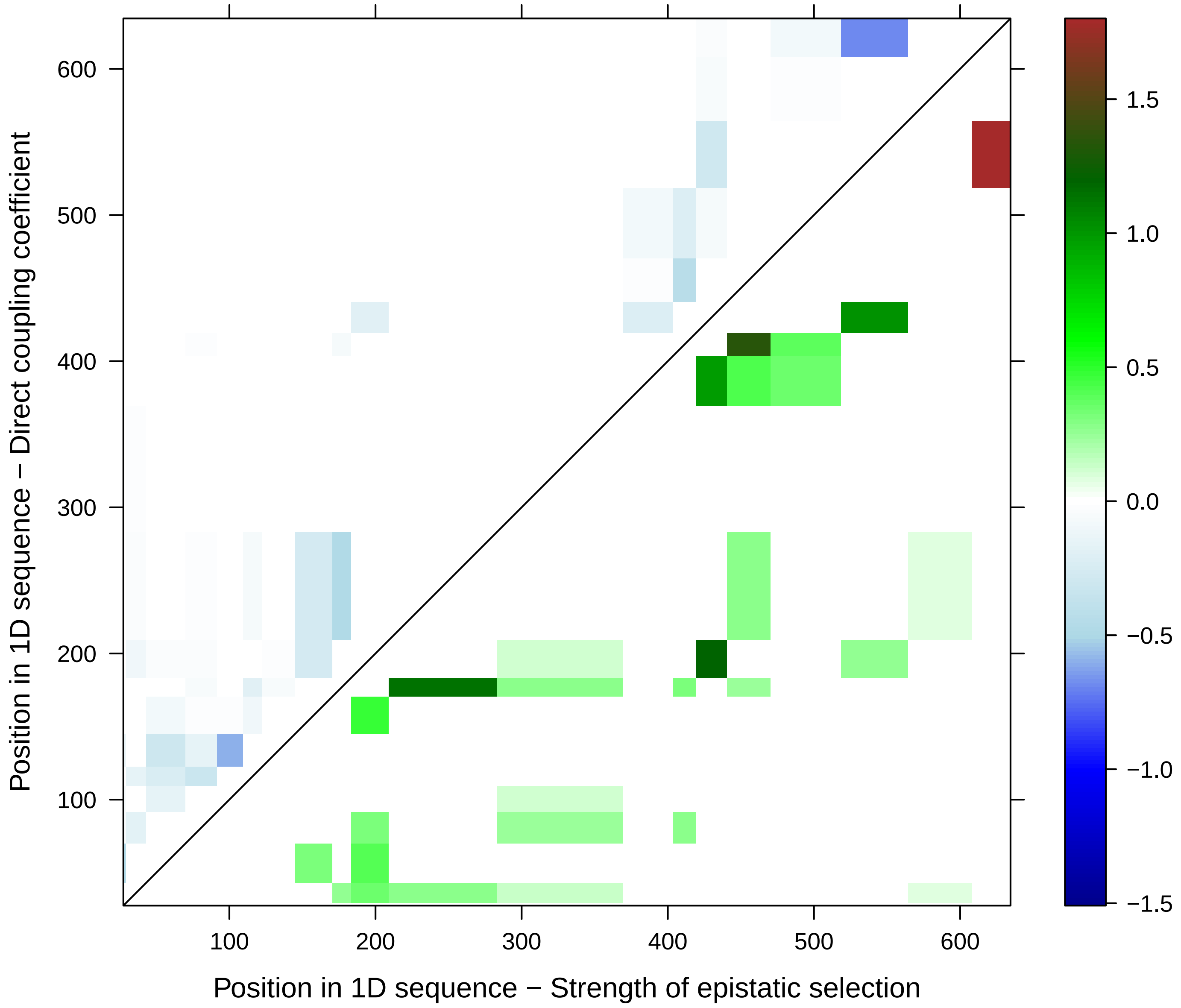

Figure M: Same as Figure J, but computed only from sequences from buffalo 44 (culled at 400 dpi).

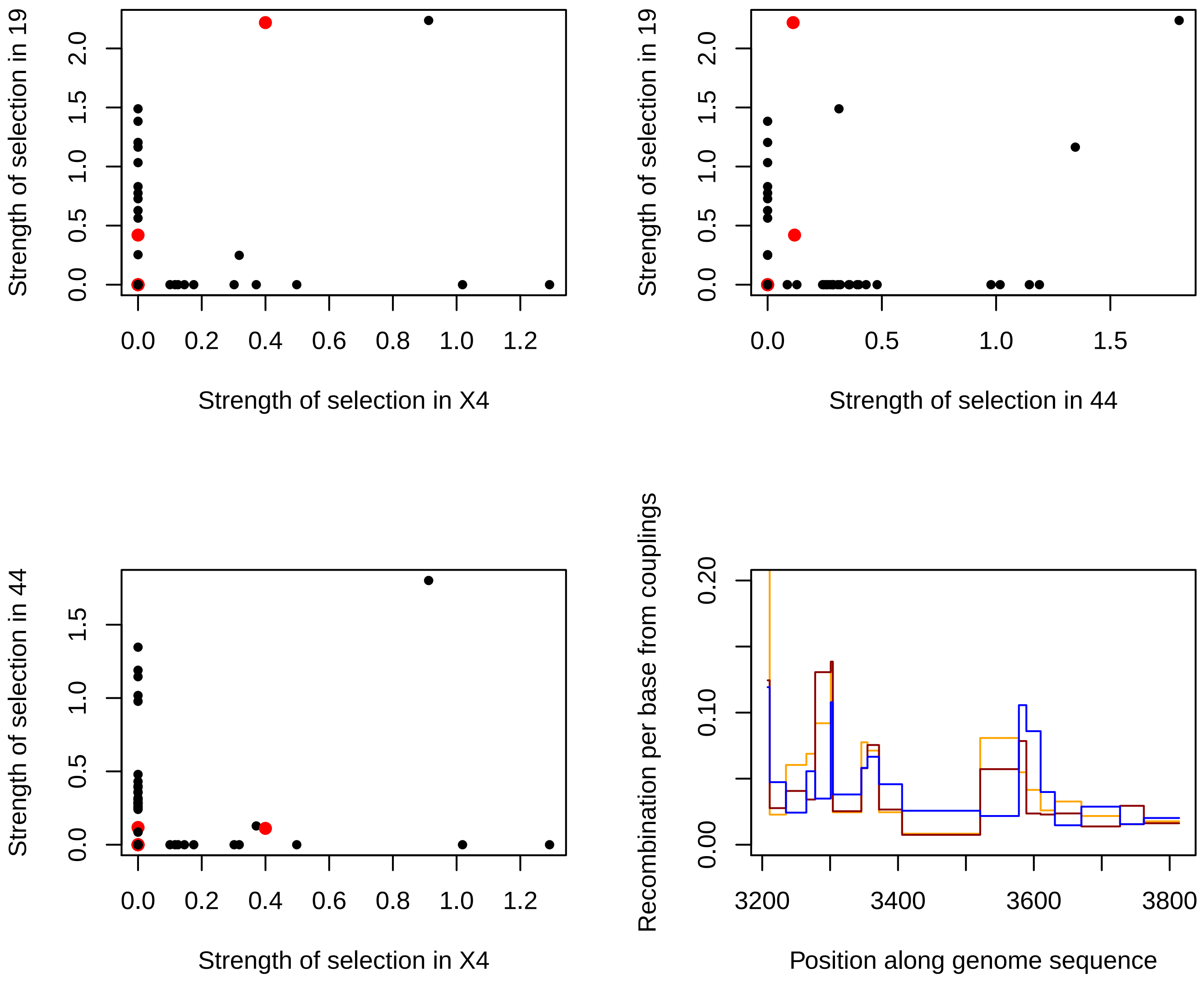

Figure N: Top, and bottom left: comparisons of the inferred strength s' of epistatic selection coefficients between sequences from different infected animals (buffalo 19, X4 and 44). Bottom right: local recombination rates inferred from DCA couplings.

and the normalised LD is then defined by $D' = D/q(1-q)$

$$D'_{ij} = \frac{J^{-1}_{ij}}{16q(1-q)} \tag{S27}$$

which implies that up to an irrelevant constant, we obtain a focal relation for the direct couplings:

$$\hat{J}_{ij} = D'^{-1}_{ij} \tag{S28}$$

This equation is the basis for the inference of direct couplings from LD values.

This DCA approach is particularly interesting because under the assumptions that there is no epistasis but variants are coupled by physical linkage only, i.e. $D'_{ij} = e^{-\sum_{k=i}^{j-1} R_{k,k+1}}$, we retrieve precisely the natural result that we would like to obtain: $\hat{J}_{ij}$ has non-zero components only between consecutive variants, i.e. the impact of physical linkage is restricted to nearest neighbours and no spurious interactions are inferred. The value of coupling coefficients between consecutive variants is inversely related to the recombination rate between the variants as

$$\hat{J}_{i,i+1} = \hat{J}_{i+1,i} = -\frac{1}{2\sinh(R_{i,i+1})} \tag{S29}$$

$$\hat{J}_{i,j} = 0 \quad , \quad |i-j| > 1 \tag{S30}$$

This shows that the approach is well suited for the analysis of epistatic interactions in recombining sequences. The (irrelevant) diagonal components are $\hat{J}_{i,i} = \frac{\sinh(R_{i,i+1})+\sinh(R_{i-1,i})}{2\sinh(R_{i-1,i})\sinh(R_{i,i+1})}$ for $i \neq 1, s$, $\hat{J}_{1,1} = \frac{e^{R_{1,2}}}{e^{R_{1,2}} - e^{-R_{1,2}}}$ and $\hat{J}_{s,s} = \frac{e^{R_{s-1,s}}}{e^{R_{s-1,s}} - e^{-R_{s-1,s}}}$.

In practice, estimating the inverse of the LD matrix (S28) is extremely noisy and requires some assumptions of sparseness for the interactions. We employ a graphical LASSO regularization approach, which is particulary suitable for Gaussian models [9], for the computation of the inverse covariance matrix choosing a regularization parameter $\rho = 0.2$.

First, it is interesting to see that equation (S29) of the DCA approach can be used to reconstruct correctly the shape of the recombination profile along VP1 (bottom right panel of Figure N). However, the absolute values across different buffaloes do not correspond to the one we inferred from LD; this relative insensitivity to the absolute values of recombination coefficients appears to be a limitation of the DCA approach, as illustrated by the results of non-epistatic simulations in Figure O.

The results for the coupling coefficients are shown in Figure J, K, L and M. A comparison between coupling coefficients and strengths of selection is shown in Figure P. Many pairs with non-zero coupling coefficients are actually inferred to be non-interacting or weakly interacting by our heuristic approach. However, interacting pairs detected by both approaches show a remarkable correlation in their inferred interaction strengths, confirming the validity of the two approaches.

S10 Epistasis versus RNA and protein structure

To assess if there is selection at the protein level, we looked for an excess in epistatic interactions detected between non-synonymous variants compared to the interactions between synonymous variants. Such an excess in the strength of non-synonymous epistatic interactions is clearly visible in the bottom panels of Figure P. To prove the significance of this effect, we compare the median interaction strength s' for interactions involving only non-synonymous variants and for interactions involving only synonymous ones via one-tailed Mann-Whitney U-test.

We find that the results across all buffaloes are significant using our heuristic pairwise approach (p=0.005 while combining p-values by Fisher's method and p=0.006 by Stouffer's method). Even

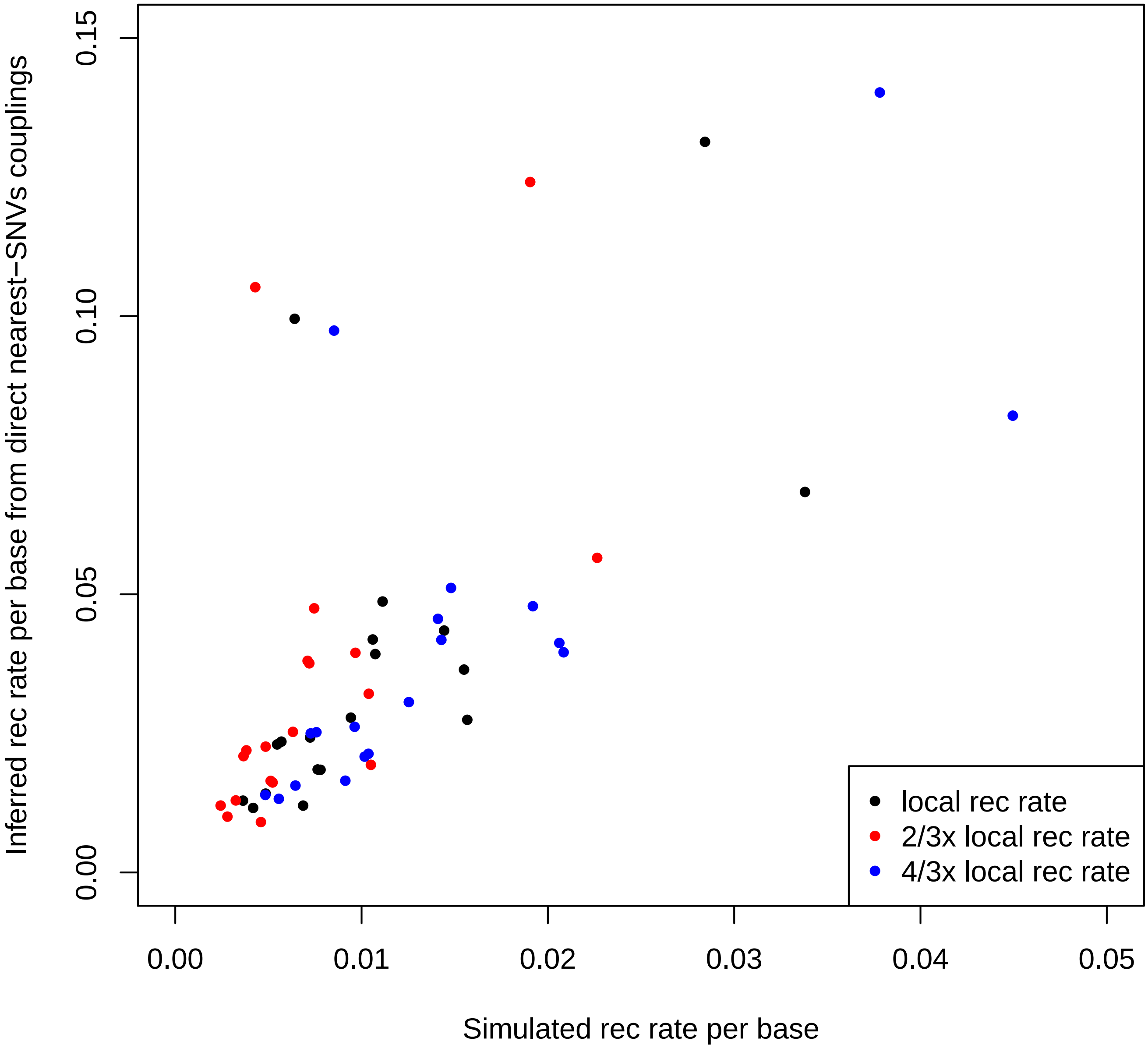

Figure O: Simulations of LD values without epistatic interactions, and of the corresponding recombination rates inferred from DCA. Local recombination rates for the simulations were chosen to correspond to the ones inferred for all VP1 sequences in this paper, or to $2/3$ or $4/3$ of their value.

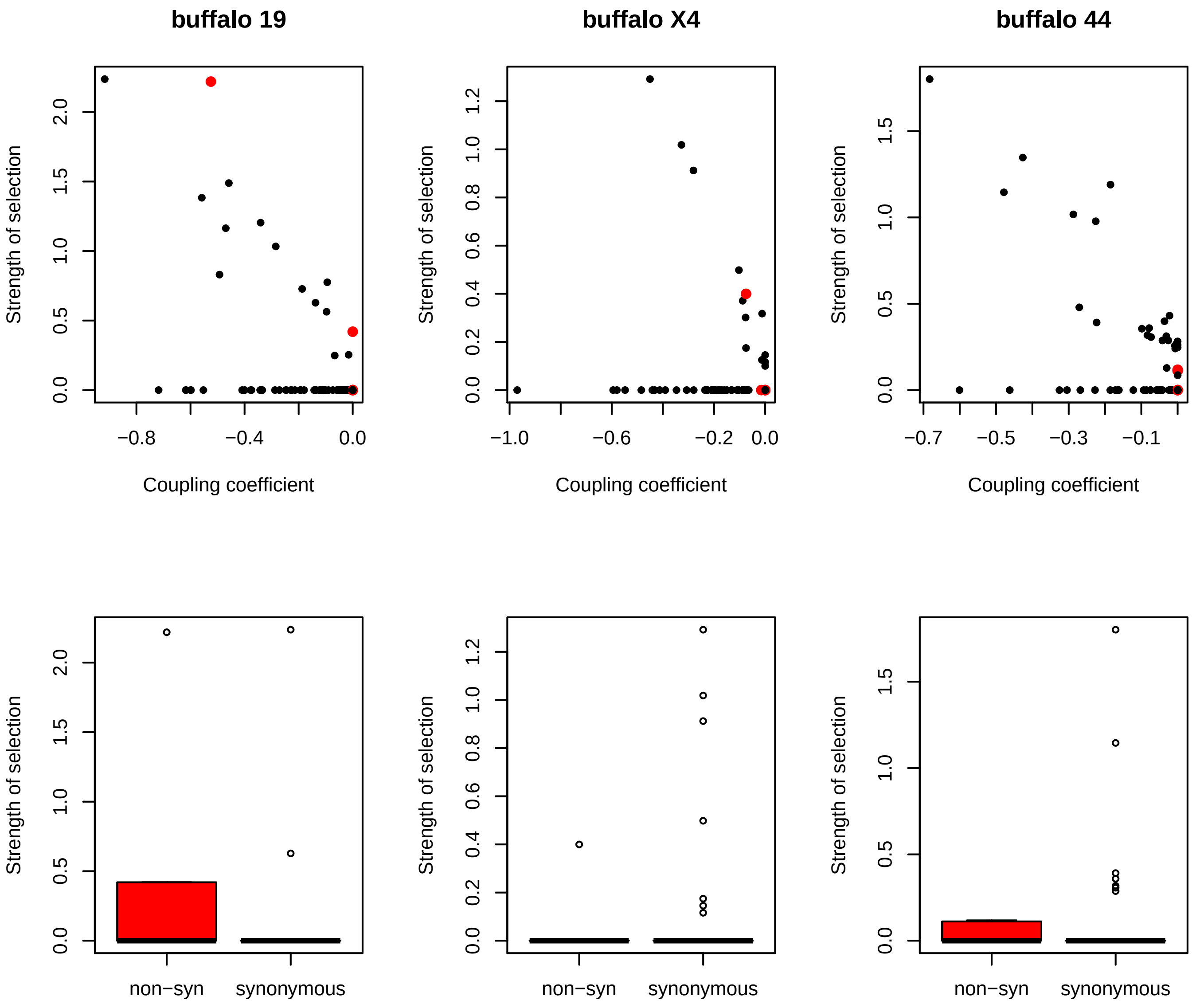

Figure P: Top: comparison between measures of the strength of epistatic interactions (inferred selection coefficients vs DCA coupling coefficients). Buffaloes 19 and X4 were culled at 35 dpi, buffalo 44 at 400 dpi. Red dots represent pairs of non-synonymous variants. Bottom: boxplots illustrating the distribution of the strength of epistatic interactions between non-synonymous variants versus strength of selection between synonymous variants

using the noisier DCA-based approach, the couplings between non-synonymous mutations tend to be significantly stronger (p=0.05 by Fisher's method and p=0.03 by Stouffer's method). This proves that some of the epistatic interactions detected here act at the protein level. It also confirms that our approaches to detect epistatic interactions are sensitive enough to capture some of the information present in the data.

For a functional interpretation of epistatic interactions among the inter-swarm variants, we analysed their localisation within the RNA secondary structure of FMDV, as well as the localisation of non-synonymous variants in the capsid.

There are four non-synonymous variants in the VP1-coding region, corresponding to four aminoacid mutations: H18Y, K49R, A99T and E179V. The localisation of these four non-synonymous variants in the capsid is illustrated in Figure 8 based on the capsid structure of the SAT1 isolate SAT1/BOT/1/68 [10, 11]. Two of these aminoacids are exposed (99 and 179), and two are close to known or suspected epitopes (49 and 99, see [12]). Three of these variants are characterised by epistatic interactions, illustrated in Figure 8 in the Main Text. The interaction between H18Y and A99T is particularly strong and is detected in all buffaloes, while the interaction between K49R and A99T is weaker and detected only in sequences from two of the animals. Note that aminoacid 99 is exposed while the interacting residue 18 is fully buried, hence this epistatic interaction could be a compensatory interaction related to the stability of the VP1 protein and the capsid structure

The other interactions are likely to be related to the RNA secondary structure of the virus. The free energy of the RNA secondary structure has been computed using rnafold from ViennaRNA [13] for all Sanger sequences from buffalo tissues. The secondary structure corresponding to the minimum free energy for the consensus sequence of the inoculum and the localisation of inter-swarm SNVs are shown in Figure Q. Most of these variants are not localised within a stem or lie on the terminal bases of a stem, hence are not expected to play a major role in the stability of RNA secondary structure.

The impact of the two swarms, the recombinants and the intra-swarm variants on the free energy of the folded RNA structure have been estimated by ANOVA based on three variables: (i) the relative contribution to each swarm to the sequence, (ii) the diversity in the recombinant origin of the sequence - i.e. the probability that two random variants in the sequence are derived from the same swarm - and (iii) the number of intra-swarm minor variants. Both swarms seem to have similar minimum free energy (about -245 kcal/mol), but recombinants tend to be surprisingly slightly more stable (up to -1.5 kcal/mol, p=0.02) while intra-swarm variants significantly reduce stability ($+0.25$ kcal/mol for each extra intra-swarm mutation in the sequence, p=0.017) as expected if they would be dominated by slightly deleterious mutations of low frequency. These results and the small values of the differences in free energy suggest that RNA stability is not the main cause of the decrease in fitness of the recombinants observed in our experiment. This agrees with the above observation on the localisation of inter-swarms SNVs.

The strongest inferred interaction between synonymous variants is shown in Figure Q. We could not find any functional interpretation for this interaction. Since it involves the last mutation at the 3' end of the sequenced region, it is also possible that this interaction is actually an artifact due to the indirect effect of an even stronger epistatic interaction with some variant outside the sequenced region.

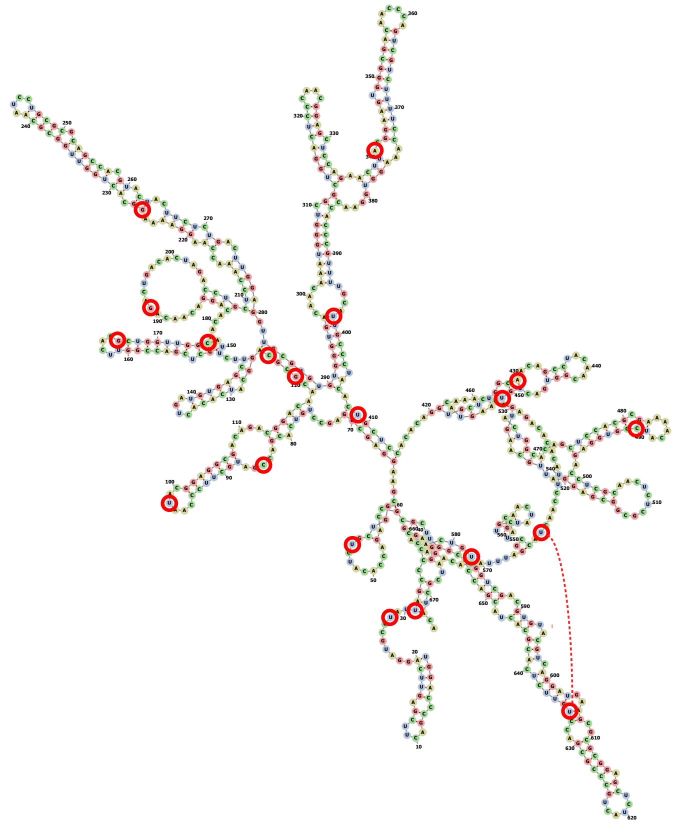

Figure Q: Localisation of inter-swarm variants in the RNA secondary structure inferred from ViennaRNA [13] using the consensus of viral sequences from buffaloes. All variants that are polymorphic both in the inoculum and within buffaloes are highlighted by red circles. The strongest epistatic interaction inferred among synonymous variants is illustrated by a dotted lines.

S11 Evolutionary consequence of recombination in the FMDV capsid

Recombination as a leading force in generating within-host diversity

One of the features of RNA viruses such as FMDV is the formation of viral swarms and quasi-species. This is a consequence of their high mutation rate [14]. The actual amount of genetic diversity within the swarm depends on the length of the infection, on the transmission bottlenecks and on the equilibrium between mutation, recombination and the selective pressures on the quasi-species. Genetic diversity in quasi-species has functional roles that are not fully understood: among others, it may increase pathogenicity and adaptation to specific tissues [15, 14].

The rates of intra-host recombination observed in this experiment suggest that recombination could be a leading force in generating genetic diversity in the swarms. In fact, a simple calculation using the substitution rates known from the literature shows that in an infected buffalo only about half of the FMDV sequences at the end of the acute infection phase would differ from the inoculum, most of them by a single nucleotide mutation; on the other hand, according to the rates observed here, most sequences would be recombinants of 4-5 viruses in the swarm. Most of these recombination events would be between almost identical viruses and do not lead to new haplotypes, but a sizeable fraction of events would create new haplotype combinations separated from the inoculum by multiple mutations, therefore enhancing the breadth of the swarm in genotype space while alleviating the mutational load by disentangling the fate of beneficial and deleterious mutations [16]. This effect is illustrated in Figure S18A.

The multi-swarm structure of our experiment offers some indirect evidence to back these observations. We perform a further analysis on the viral sequences from buffalo tissues, ignoring the intermediate-frequency SNVs that distinguish the two swarms, and focus on the low-frequency SNVs within each swarm. The corresponding genetic diversity is a good representation of the potential intra-host diversity of a single quasi-species. For each buffalo, in the absence of recombination and assuming that multiple mutations play a minor role, the haplotypic diversity of the sample (defined as the number of haplotypes [17]) should be equal to the number of these SNVs plus one, but it turns out to be systematically higher. In fact, for the swarms infecting the two individuals culled at 35 dpi, recombination could account for about 31% and 28% of the haplotypic diversity in VP1, respectively. In the animal culled after one year of persistent infection, the haplotypic diversity in VP1 is close to saturation (i.e. almost all sequences are different haplotypes) and 75% of it could be attributed to recombination. This rise in the role of recombination in time is consistent with the observed increase in recombination between swarms and supports the persistent replication of the virus even in the carrier state.

Reduction in the fitness of recombinants during co-infections

The dynamics of recombination are different when an animal is co-infected by multiple strains, as in our experiment. As discussed before, combinations of alleles belonging to the same strain often have higher fitness even within host, since these combinations have already co-evolved through a range of selective pressures for infectivity and stability and are already adapted. This is a case of positive epistasis between these variants.

Recombination causes the disruption of these beneficial coevolved genetic interactions [18, 19]. Hence, the within-host selective pressures due to epistatic interactions tend to reduce the number of recombinants and therefore the effective rate of recombination [20]. This effect is visible even in our data. From Figure 7, the number of recombinants with two VP1 blocks of different origin is suppressed by an order of magnitude with respect to the naive expectation based on the inferred

rates. From a back-of-the-envelope computation, the suppression factor can be estimated as $e^{-s \cdot t}$ where s is the fitness disadvantage of recombinants within the host and t is the number of viral generations since the beginning of the infection. Since a replication cycle of FMDV is completed in a few hours, we estimate that intra-host epistatic interactions in VP1 are quite strong, with selection coefficients up to $s \approx 0.1$ per generation.

Given the further selective constraints on infectivity and transmissibility, recombination between different strains could easily generate recombinants with suboptimal combinations of variants not only for within-host growth, but for between-host transmission as well, as discussed in the next section. That would then significantly reduce the probability of observing these recombinants in other hosts. This effect increases with the amount and strength of epistatic interactions, but it occurs even for weak epistasis among many variants [21].

The interplay of recombination and epistasis results in the exchange of short sequences

At high recombination rates, there would be a number of recombinants that are almost identical to one of the original strains but for short sequence stretches coming from the other strain. The role of recombination is to mediate these exchanges of short fragments that tend to have a limited impact on fitness and could even form new beneficial allelic combinations. Sequences derived from such exchanges may be transmitted and infect other animals, hence playing a role in the generation of capsid genetic diversity at epidemiological scales. This phenomenon is illustrated in Figure S18B. These exchanges of short recombinant fragments would be almost undetectable from phylogenetic analyses since they would likely be attributed to convergent point mutations, but would actually be originating from co-infection and recombination.

Some indirect evidence for this phenomenon can be found in the samples obtained via tonsil swabs and probangs from four buffaloes involved in the experiment, all of them infected with the same inoculum. These samples show almost no internal variability, while at the consensus level, their sequences correspond to recombinants of the two initial swarms [2]. In these samples, we actually observe such exchanges of short fragments, most of them being about a few tens of bases long. In fact, these recombinant sequences show a clear asymmetry in the amount of bases derived from each swarm: the average contribution of the major swarm of the inoculum is less than 20% and it is scattered in small fragments with a median estimated length of 27 bases, much smaller than the median length of ~ 130 expected for randomly located recombination breakpoints ($p < 10^{-8}$ by Mann-Whitney U-test). Each of these fragments contains on average 1-2 swarm-specific variants.

This recombination-mediated exchange of short fragments is a potentially relevant mechanism for genetic exchange between capsid sequences of different FMDV strains. Its evolutionary role could mimic what occurs in non-structural proteins, where the exchange of large fragments and the “mosaic” structure of the genome play an important role in long-term viral evolution by spreading genetic variability across different serotypes.

S12 Mismatch between intra- and inter-host recombination rates

Within-host recombination between different FMDV strains during co-infections sometimes results in sequences of high fitness containing large recombinant fragments, which can be transmitted and are able infect other animals, hence playing a role in the long-term evolution of the virus.

These recombination events can also be inferred at phylogenetic scales, i.e. from FMDV sequences collected from different animals and locations. In fact, in the presence of recombination different regions of the genome might have different genealogical trees. If the recombinant fragments are large enough, this signal can be detected in phylogenetic analyses. Such analyses were

performed in the past to infer FMDV recombination breakpoints from phylogenetic incompatibilities [22, 23].

There is a clear mismatch between the within-host recombination rates observed in our experiment and the much lower recombination rates inferred from phylogenetic analyses. As an example, the number of recombination events in the capsid region inferred in [22] for the whole FMDV phylogeny is similar to the number of intra-host events that we observe after one year of persistent infection in a single individual! In addition, while our findings imply that structural proteins recombine less than non-structural ones located in flanking regions in the genome, this difference in recombination rates appears to be much less extreme than the one observed on a phylogenetic scale. Genome-wide differences in the patterns of within-host versus phylogenetic recombination have been studied in a related paper [24], yielding qualitatively similar results.

However, there is another key difference between this study and previous studies. Previous phylogenetic investigations focused on recombination between highly divergent sequences: in [23] only inter-serotype recombination events were considered, while in [22], parent sequences belonged to different serotypes in about 70% of the detected events. In contrast, the two main swarms studied here are very similar and belong to the same topotype. This suggests that divergence-dependent effects that suppress recombination on broad scales could be responsible for the mismatch.

Artefacts like biased detection in phylogenetic analyses could partially explain this result. Inference of recombination by phylogenetic methods depends on the resolution of the tree and the similarity of recombining sequences. Inferring recombination between similar sequences is very difficult, since the trees generated by these events are very similar to each other [25] and there are not enough mutations to resolve the recombining branches. In particular, recombination between very close sequences in the phylogenetic tree is hardly detectable. This affects structural and non-structural proteins in a similar way, since it depends only on the local molecular clock. Hence, this cannot be the only reason for the mismatch.

Cross-immunity, population structure and epistasis suppress recombination

There are several other genetic and epidemiological factors that can suppress FMDV recombination in endemic and epidemic contexts. Some of these factors could have a stronger impact on structural proteins than on non-structural ones. The mismatch between intra-host and phylogenetic recombination rates offers new opportunities to study these factors.

One of these factors is cross-immunity. The effective rate of recombination is proportional to the rate of co-infections, since co-infection of the same animal/cell by two different strains is a necessary condition for recombination to occur via template switching [26]. The probability of co-infections depends on the ability of the second strain to escape the immune response induced by the first strain, i.e. on the cross-immunity between strains. Cross-immunity depends mostly on capsid proteins [27] (since they are exposed to the immune system) and it tends to decrease with increasing divergence between the capsid sequences of the two strains, hence suppressing recombination between closely related strains only.

Another related factor is the co-circulation of lineages. Geographical separation of lineages could reduce the probability of co-infection and recombination, since the spatial co-existence of different FMDV lineages in the same area is a prerequisite for non-trivial recombination [26]. However, spatial patterns of FMDV are complex and depend on the endemic/epidemic system considered, so the importance and the actual role of this effect is difficult to estimate.

Finally, selection for infectivity and transmissibility would enhance the role of epistatic genetic interactions between variants of well-adapted strains. Such interactions have been recently detected in phylogenetic studies of influenza A [28, 29]. While epistatic interactions in infectivity and between-host transmissibility could be present among all FMDV protein-coding regions, it is reasonable to

expect stronger selection in the capsid, due to the amount of structural interactions between capsid proteins and the interplay between opposite selective pressures from stability and from the immune system of the host.

The pattern of suppression of recombination due to these constraints is illustrated in Fig RC using a simple model of cross-immunity and epistatic incompatibilities. For most reasonable values of cross-immunity and epistatic parameters, the model predicts a strong suppression of the recombination rate in structural proteins. The suppression can be of several orders of magnitude, which would explain the near absence of recombination events in capsid on a phylogenetic scale.

Recombination, epistasis and speciation in FMDV

Interestingly enough, it was suggested in [28] that the suppression of recombination due to epistatic interactions could act as a mechanism for viral speciation, playing a similar role as hybrid incompatibilities in the classical Dobzhansky-Muller model of speciation [30, 31]. Sympatric speciation is known to occur in viruses [32, 33] and could be caused precisely by the dependence of epistasis and suppression on the amount of divergence between sequences. In the context of FMDV and other picornaviruses, speciation appears to be inhibited by capsid-swapping [22] and frequent recombination events in the genomic regions coding for non-structural proteins. However, epistatic interactions could lead to a genetic separation between incompatible capsid sequences, being therefore the causal factor in the emergence of different serotypes.

References

[1] Maree F, de Klerk-Lorist LM, Gubbins S, Zhang F, Seago J, Pérez-Martín E, et al. Differential persistence of foot-and-mouth disease virus in African buffalo is related to virus virulence. Journal of virology. 2016;90(10):5132–5140.

[2] Cortey M, Ferretti L, Pérez-Martín E, Zhang F, de Klerk-Lorist LM, Scott K, et al. Persistent infection of African buffalo (Syncerus caffer) with Foot-and-Mouth Disease Virus: limited viral evolution and no evidence of antibody neutralization escape. Journal of Virology. 2019;doi:10.1128/JVI.00563-19.

[3] Katoh K, Standley DM. MAFFT multiple sequence alignment software version 7: improvements in performance and usability. Molecular biology and evolution. 2013;30(4):772–780.

[4] Hartl DL, Clark AG, Clark AG. Principles of population genetics. vol. 116. Sinauer associates Sunderland; 1997.

[5] Zapata C, Alvarez G, Carollo C. Approximate variance of the standardized measure of gametic disequilibrium D'. American journal of human genetics. 1997;61(3):771.

[6] Strutz T. Data fitting and uncertainty: A practical introduction to weighted least squares and beyond. Vieweg and Teubner; 2010.

[7] Weigt M, White RA, Szurmant H, Hoch JA, Hwa T. Identification of direct residue contacts in protein–protein interaction by message passing. Proceedings of the National Academy of Sciences. 2009;106(1):67–72.

[8] Baldassi C, Zamparo M, Feinauer C, Procaccini A, Zecchina R, Weigt M, et al. Fast and accurate multivariate Gaussian modeling of protein families: predicting residue contacts and protein-interaction partners. PloS one. 2014;9(3):e92721.

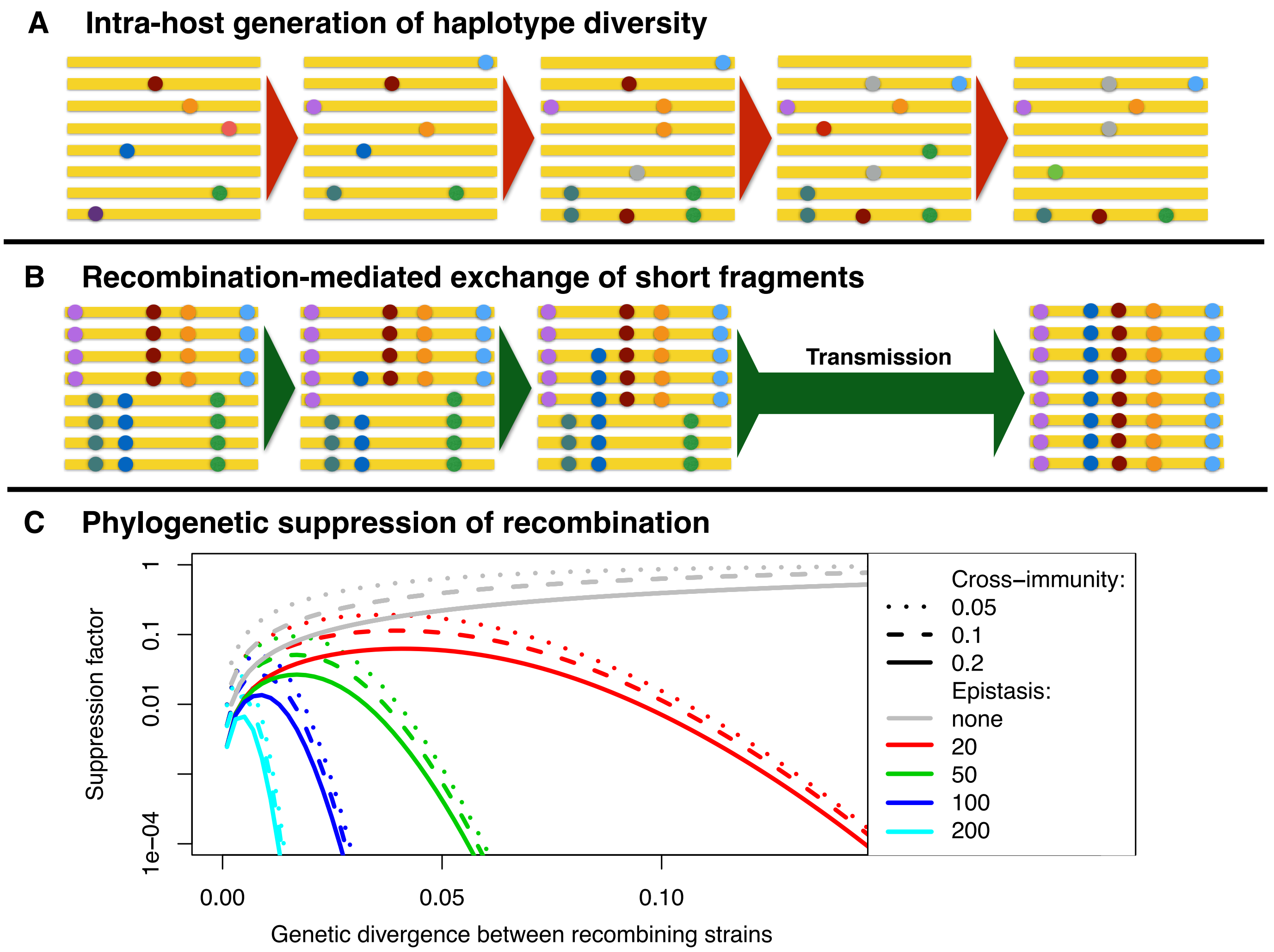

Figure R: **Illustration of the evolutionary consequences of recombination.** A: Evolution of a swarm viral sequences (in yellow) and variants therein (coloured dots) under mutation and recombination. Intra-host recombination cannot generate new mutations in the swarm, but it generates new haplotypes from existing mutations and it disentangles the evolutionary fate of different mutations, reducing the effect of deleterious mutations and the mutational load. B: During co-infections by divergent strains, recombination may cause the exchange of short sequence fragments between strains. If the resulting viral strains are similar to the original ones, they may be transmitted despite the selective pressures against recombinants, as illustrated. C: Plot of the predicted reduction in combination in structural proteins at phylogenetic scales, due to cross-immunity and epistatic interactions, shown as a function of the genetic divergence (i.e. Hamming distance per base) between recombining sequences. Cross-immunity is modelled as an exponentially decreasing function of the divergence d between strains, following an approach used for influenza [34]. Assuming that cross-immunity acts between lineages with divergence less than d_{ci}, the reduction in recombination corresponds to the reduction in co-infection $1-e^{-d/d_{ci}}$. Epistasis is modelled after [30] as a decrease in viral growth rate proportional to the number of pairwise interactions disrupted by recombination, leading to a recombination suppression factor $e^{-s_e^2 d^2}$ where d is the genetic divergence and the epistatic coefficient s_e is related to the strength and number of epistatic interactions. Assuming that the strength of epistasis among different capsid protein-coding sequences is is comparable to the one observed within host between the two blocks of VP1, a conservative estimate for this coefficient could be around $s_e \sim 20$.

[9] Friedman J, Hastie T, Tibshirani R. Sparse inverse covariance estimation with the graphical lasso. Biostatistics. 2008;9(3):432–441.

[10] Carrillo C, Tulman E, Delhon G, Lu Z, Carreno A, Vagnozzi A, et al. Comparative genomics of foot-and-mouth disease virus. Journal of virology. 2005;79(10):6487–6504.

[11] Adams P, Lea S, Newman J, Blakemore W, King A, Stuart D, et al. The Structure of Foot-and-Mouth Disease Virus Serotype Sat1; 2010.

[12] Mukonyora M. The in silico prediction of foot-and-mouth disease virus epitopes on the South African Territories SAT1, SAT2 and SAT3 serotypes [M.Sc. thesis]. University of South Africa; 2015.

[13] Lorenz R, Bernhart SH, Zu Siederdissen CH, Tafer H, Flamm C, Stadler PF, et al. ViennaRNA Package 2.0. Algorithms for Molecular Biology. 2011;6(1):26.

[14] Domingo E, Sheldon J, Perales C. Viral quasispecies evolution. Microbiology and Molecular Biology Reviews. 2012;76(2):159–216.

[15] Lauring AS, Andino R. Quasispecies theory and the behavior of RNA viruses. PLoS pathogens. 2010;6(7):e1001005.

[16] Xiao Y, Dolan PT, Goldstein EF, Li M, Farkov M, Brodsky L, et al. Poliovirus intrahost evolution is required to overcome tissue-specific innate immune responses. Nature communications. 2017;8(1):375.

[17] Gregori J, Perales C, Rodriguez-Frias F, Esteban JI, Quer J, Domingo E. Viral quasispecies complexity measures. Virology. 2016;493:227–237.

[18] Martin DP, Van der Walt E, Posada D, Rybicki EP. The evolutionary value of recombination is constrained by genome modularity. PLoS genetics. 2005;1(4):e51.

[19] Monjane AL, Martin DP, Lakay F, Muhire BM, Pande D, Varsani A, et al. Extensive recombination-induced disruption of genetic interactions is highly deleterious but can be partially reversed by small numbers of secondary recombination events. Journal of virology. 2014;88(14):7843–7851.

[20] Franklin I, Lewontin R. Is the gene the unit of selection? Genetics. 1970;65(4):707–734.

[21] Neher RA, Shraiman BI. Competition between recombination and epistasis can cause a transition from allele to genotype selection. Proceedings of the National Academy of Sciences. 2009;106(16):6866–6871.

[22] Heath L, Van Der Walt E, Varsani A, Martin DP. Recombination patterns in aphthoviruses mirror those found in other picornaviruses. Journal of Virology. 2006;80(23):11827–11832.

[23] Jackson A, O'neill H, Maree F, Blignaut B, Carrillo C, Rodriguez L, et al. Mosaic structure of foot-and-mouth disease virus genomes. Journal of General Virology. 2007;88(2):487–492.

[24] Ferretti L, Di AN, Singer B, Lasecka-Dykes L, Logan G, Wright C, et al. Within-Host Recombination in the Foot-and-Mouth Disease Virus Genome. Viruses. 2018;10(5).

[25] Ferretti L, Disanto F, Wiehe T. The effect of single recombination events on coalescent tree height and shape. PloS one. 2013;8(4):e60123.

[26] Worobey M, Holmes EC. Evolutionary aspects of recombination in RNA viruses. Journal of General Virology. 1999;80(10):2535–2543.

[27] Bøtner A, Kakker NK, Barbezange C, Berryman S, Jackson T, Belsham GJ. Capsid proteins from field strains of foot-and-mouth disease virus confer a pathogenic phenotype in cattle on an attenuated, cell-culture-adapted virus. Journal of General Virology. 2011;92(5):1141–1151.

[28] Villa M, Lässig M. Fitness cost of reassortment in human influenza. PLoS pathogens. 2017;13(11):e1006685.

[29] White MC, Lowen AC. Implications of segment mismatch for influenza A virus evolution. Journal of General Virology. 2017;.

[30] Orr HA. The population genetics of speciation: the evolution of hybrid incompatibilities. Genetics. 1995;139(4):1805–1813.

[31] Gavrilets S. Hybrid zones with Dobzhansky-type epistatic selection. Evolution. 1997;51(4):1027–1035.

[32] Kitchen A, Shackelton LA, Holmes EC. Family level phylogenies reveal modes of macroevolution in RNA viruses. Proceedings of the National Academy of Sciences. 2011;108(1):238–243.

[33] Meyer JR, Dobias DT, Medina SJ, Servilio L, Gupta A, Lenski RE. Ecological speciation of bacteriophage lambda in allopatry and sympatry. Science. 2016; p. aai8446.

[34] Łuksza M, Lässig M. A predictive fitness model for influenza. Nature. 2014;507(7490):57.
